# Interplay of FXN expression and lipolysis in white adipocytes plays a critical role in insulin sensitivity in Friedreich’s ataxia mouse model

**DOI:** 10.1038/s41598-024-71099-7

**Published:** 2024-08-27

**Authors:** Lin Wu, Fei Huang, Lu Yang, Liu Yang, Zichen Sun, Jinghua Zhang, Siyu Xia, Hongting Zhao, Yibing Ding, Dezhi Bian, Kuanyu Li

**Affiliations:** 1 Jiangsu Key Laboratory of Molecular Medicine, Medical School, Nanjing University, Nanjing, 210093 People’s Republic of China; 2 Endocrinology Department, Yancheng First People’s Hospital, Affiliated Hospital of Medical School, Nanjing University, Yancheng, 224000 People’s Republic of China

**Keywords:** Frataxin, PPARγ, Lipolysis, Insulin sensitivity, Adipose tissue, Mechanisms of disease, Metabolism

## Abstract

Frataxin (FXN) is required for iron-sulfur cluster biogenesis, and its loss causes the early-onset neurodegenerative disease Friedreich ataxia (FRDA). Loss of FXN is a susceptibility factor in the development of diabetes, a common metabolic complication after myocardial hypertrophy in patients with FRDA. The underlying mechanism of FXN deficient-induced hyperglycemia in FRDA is, however, poorly understood. In this study, we confirmed that the FXN deficiency mouse model YG8R develops insulin resistance in elder individuals by disturbing lipid metabolic homeostasis in adipose tissues. Evaluation of lipolysis, lipogenesis, and fatty acid β-oxidation showed that lipolysis is most severely affected in white adipose tissues. Consistently, FXN deficiency significantly decreased expression of lipolytic genes encoding adipose triglyceride lipase (Atgl) and hormone-sensitive lipase (Hsl) resulting in adipocyte enlargement and inflammation. Lipolysis induction by fasting or cold exposure remarkably upregulated FXN expression, though FXN deficiency lessened the competency of lipolysis compared with the control or wild type mice. Moreover, we found that the impairment of lipolysis was present at a young age, a few months earlier than hyperglycemia and insulin resistance. Forskolin, an activator of lipolysis, or pioglitazone, an agonist of PPARγ, improved insulin sensitivity in FXN-deficient adipocytes or mice. We uncovered the interplay between FXN expression and lipolysis and found that impairment of lipolysis, particularly the white adipocytes, is an early event, likely, as a primary cause for insulin resistance in FRDA patients at later age.

## Introduction

Friedreich ataxia (FRDA) is the most common single-gene autosomal recessive ataxia disease, caused by frataxin (*FXN*) gene mutation, with a prevalence estimated at about 1 to 50,000 and a calculated carrier frequency of ≈ 1 into 120 in Caucasian populations^1^. It is a multisystem disorder affecting the central and peripheral nervous systems, myocardium, and endocrine pancreas. Diabetes is a common metabolic complication in FRDA patients^2,3^, with incidence rates between 8 and 32%^4,5^. This variability partially relies on the differences in diagnostic criteria and the disease stage. In an earlier review of a series of fatal FRDA cases, the prevalence of clinically diagnosed diabetes was 23%^6^. When counting FRDA patients without a pre-existing clinical diagnosis of diabetes, 49% of participants had impaired fasting glucose and/or impaired glucose tolerance, and 12% had diabetes^5^, illustrating that this is a prevalent metabolic complication in FRDA patients. Both insulin deficiency and insulin resistance have been reported^3,5,7^.

By 2025, more than 300 million people are expected to have type 2 diabetes (T2D) as a complication of obesity^8^. The leading cause of T2D is obesity-driven insulin resistance (IR) in the liver, skeletal muscle, and white adipose tissue (WAT), combined with impaired insulin secretion by pancreatic β-cells to overcome IR after a long period of over nutrition^9^. WAT is a multifactorial tissue and plays a critical role in energy homeostasis control. Excess energy is usually stored as cytosolic triglycerides (TAGs)-rich lipid droplets in the adipose tissue. Mobilizing this energy involves a catabolic process called adipose lipolysis. Intracellular lipolysis is catalyzed by lipases to hydrolyze TAGs into glycerol and free fatty acids (FFAs) in three steps. TAGs are hydrolyzed first by adipose triglyceride lipase (ATGL, encoded by *Pnpla2*) into diacylglycerol (DAG) and FFAs, second by hormone-sensitive lipase (HSL, encoded by *Lipe*) from DAG into monoacylglycerol (MAG) and FFAs, and last by monoglyceride lipase (MGL, encoded by *Mgll*) from MAG into glycerol and FFAs. Among the whole process, HSL exhibits broad substrate specificity with highest activity against DAGs and cholesteryl esters (CEs), followed by TAGs, MAGs, retinyl esters and short-chain acyl esters, suggesting its broad impact in lipid metabolism. Inadequate lipolysis or accumulated TAGs aggravate numerous abhorrent processes, such as inflammation and insulin resistance^10^. As a result, adipocyte lipolysis requires exquisite regulation at the physiological levels indirectly by hormones such as insulin and directly related transcription factors such as peroxisome proliferator-activated receptor gamma (PPARγ to impact HSL and ATGL expression)^11^. Growing evidence has suggested that PPARγ is enriched in adipose tissue to play a critical role in adipocyte differentiation and the transactivation of ATGL and HSL^12,13^. PPARγ pathway was identified to be dysregulated in skeletal muscle and heart^14^, exhibiting increased lipogenesis in FRDA model mice and patient-derived cells^15^. PPARγ agonists leriglitazone^16^ and Azelaoyl PAF^17^ increased *FXN* expression in FRDA cellular or animal models. A study found that Fxn deficiency affected the expression of lipolysis genes in brown adipose tissue (BAT)^18^. However, the lipolysis ability in BAT had not been analyzed. Recently, it was found that lean mass is lower and body mass index is unaltered in patients with FRDA^19^.

We, therefore, studied how FXN deficiency affects lipolysis using the knockin–knockout YG8R mice that lack endogenous *FXN* but express a low-level human FXN as FRDA mouse model^20^. The Y47 strain was used as the control mice with the same genetic background as YG8R. We confirmed an essential role of FXN in lipolysis, particularly in WAT, and found an interplay between FXN expression and lipolysis. We also found that disrupted lipolysis ability in WAT is an early event resulting in insulin resistance at late age in FRDA mice. Enhancement of lipolysis upregulated FXN remarkably, suggesting lipolysis as a target for a pharmaceutical strategy for FRDA treatment.

## Materials and methods

### Animals and treatment

Y47 and YG8R mice were purchased from Jackson Laboratory. The animals were group-housed under standard conditions with a 12 h light–dark cycle and a temperature of 25 °C. The Animal Investigation Ethics Committee of Nanjing University reviewed and approved all animal experiments performed according to the Guidelines for the Care and Use of Laboratory Animals published by the National Institutes of Health, USA.

For high-fat-diet (HFD) treatment, Y47 and YG8R mice were fed an HFD (0.2% cholesterol and 20% fat) at the age of 8 weeks for 12 weeks. For Pioglitazone (PGZ) treatment, male Y47 and YG8R mice were treated with PGZ (25 mg/kg/d; oral administration by gavage) for 8 weeks at 40 weeks old.

### Cell culture and induction of adipocyte differentiation

Six-week-old male Y47 and YG8R mice were euthanized, and a stromal vascular fraction from eWAT pads was isolated, cultured, and differentiated as described previously^21^.

3T3-L1 preadipocytes (gift from Dr. Yue Zhao, Nanjing University) were cultured in DMEM (25 mM glucose) supplemented with 10% fetal bovine serum (FBS, cat# FSP500, ExCell Bio, Inc., Shanghai, China), 100 U/ml penicillin, and 100 mg/ml streptomycin at 37 °C in an atmosphere of 5% CO_2_. Standard hormone cocktails induced 3T3-L1 cells to differentiate into mature adipocytes^22^ for further experiments.

For the insulin sensitivity experiments, differential 3T3-L1 cells were treated with or without PGZ or FSK for 2 d. Then, cells were incubated with or without insulin (100 nM) for 20 min. PGZ (cat# MB1186) and FSK (cat# MB5959-1) were purchased from Meilun Biotechnology (Dalian, China).

### Lentivirus production and infection

Short hairpin RNA (shRNA) plasmids were constructed with lentiviral vector pLKO.1 (cat# 10878, Addgene, Watertown, MA) as described^23^. For lentivirus packaging, the shRNA vector was co-transfected with psPAX2 and pMD2.G into HEK293T cells. Medium containing lentiviral particles were either concentrated at 70,000×*g* for 2 h or directly aliquoted and stored at − 80 °C until use.

For lentiviral infection of 3T3-L1 adipocytes, 3T3-L1 cells were infected with lentivirus encoding scrambled shRNA or shRNA targeting gene in a medium containing 8–10 μg/ml polybrene. Cells were selected against 5 μg/ml puromycin for at least 72 h before differentiation. The sequences of shRNA used are provided (Table S1).

### Glucose uptake assays

Glucose uptake assay was performed using 2-NBDG by 2-NBDG Glucose Uptake Assay Kit (APExBIO, K2212). In brief, the cells were seeded in 96-well plates at 3000 cells/well in culture media overnight. Then, the cells were cultured with 2-NBDG (100 μg/ml) in glucose-free media with insulin (100 nM) at 37 °C for 20 min. The 2-NBDG uptake reaction was stopped by removing the media and washing the cells twice with Assay Buffer. The fluorescent intensity of the cells was detected on a microplate reader (Ex (λ) 485 nm; Em (λ) 535 nm).

### RNA extraction and reverse transcription-quantitative PCR

Total RNA was isolated from tissues or cells using TRIzol (Vazyme Biotech Co. Ltd., Nanjing, China) according to the manufacturer’s instructions. The HiScript III RT SuperMix (Vazyme Biotech Co. Ltd.) was used for reverse transcription, and qPCR analysis was performed with ChamQ Universal SYBR qPCR Master Mix (Vazyme Biotech Co. Ltd.) in Applied Biosystems 7300. 18S rRNA was used as an internal control. Experiments were repeated at least three times independently. The sequences of primers used are provided (Table S1).

### Immunofluorescent assays

3T3-L1 preadipocytes were treated with Oleic acid (OA, 300 μM). After 24 h, cells were fixed with 4% paraformaldehyde for 15 min and stained with Nile Red (5 μM) for 15 min. The stained cells were observed and photographed by confocal laser microscopy (Olympus FV3000, Tokyo, Japan).

### Glucose and insulin tolerance tests and serum biochemical measurement

Glucose tolerance tests (GTT) and insulin tolerance tests (ITT) were performed as previously described^24^. The levels of alanine aminotransferase (ALT, cat# C009-2-1), aspartate aminotransferase (AST, cat# C010-2-1), triglycerides (TAGs, cat# A110-1-1), and total cholesterol (TC, cat# A111-1-1) were measured following the manufacturer’s instructions (Nanjing Jiancheng Bioengineering Institute, Nanjing, China). The insulin level was measured by a mouse insulin detection kit (cat# EM017-48, ExCell Bio, Inc., Shanghai, China). The FFAs level was measured by the FFAs content assay kit (cat# BC0595, Solarbio Science & Technology Co. Ltd., Beijing, China).

### Histological assays

The tissues were fixed in 4% paraformaldehyde and sectioned into 2 to 7 μm thick. Hematoxylin–eosin staining (H&E staining) was performed as previously reported^25^.

For immunohistochemistry, tissue sections were incubated with anti-F4/80 (1:500). The reaction was attenuated, dehydrated, and sealed after color rendering of 3,3′-diaminobenzidin (DAB, ZSGB-BIO, Beijing, China). Cells with brownish-yellow cytoplasm were counted as positive cells.

### Lipolysis assays

Wild type, Y47 and YG8R mice were fasted for 24 h or exposed to cold (4 °C) for 6 h. Mice were sacrificed under anesthesia, and sera were extracted. Serum FFA and glycerol were determined to illustrate the lipolysis capacity^26^ and measured respectively by the FFAs content assay kit (cat# BC0595, Solarbio Science and Technology Co. Ltd., Beijing, China) and glycerol detection kit (cat#E1002, Applygen Technologies Inc, Beijing, China).

Differentiated NC and shFxn 3T3-L1 cells were incubated with phenol red-free, serum-free DMEM containing 1% FA-free BSA and 50 μM of FSK for 4 h. FFAs and glycerol released into the medium were measured using the kits described above.

### The enzymatic activities of complexes I and II and ATP content

The activities of complexes I and II were measured following the manufacturer’s protocols. Purchase information is as follows: Complex I from Abcam and Complex II from Comin Biotechnology Co. (Suzhou, Jiangsu, China). ATP content in the adipose tissue of Y47 and YG8R mice was measured using an ATP content assay kit (Beyotime Biotech).

### Western blot analysis

The harvested cells and mouse tissues were analyzed by immunoblotting as described previously^25^. The FXN antibody was validated in a previous study^23^. Information on the other antibodies used is provided (Table S2). When it is necessary to detect multiple proteins in one blot and the molecular weight of the protein is different, we cut the blotted nitrocellulose membrane according to the molecular weight and then incubate with different antibodies. When the molecular weights are very close, we ran multiple gels with the same prepared total protein samples, transfer them to nitrocellulose membranes, cut them according to molecular weights, and then incubate them with different antibodies. We used Tanon Science and Technology Co., Ltd. (Shanghai, China) ECL-plus reagent to visualize the detected proteins. Original images of blots are provided in Figs. S7–S20.

### Statistics analysis

Data were presented as mean ± SD. All the experiments were repeated more than three times independently. Student’s *t*-test or one-way analysis of variance (ANOVA) was performed using GraphPad Prism 8. Significance was considered at P < 0.05.

### Ethics approval

All protocols were approved by the Animal Investigation Ethics Committee of the Nanjing University Medical School and were performed according to the Guidelines for the Care and Use of Laboratory Animals published by the National Institutes of Health, USA. The study was also carried out in compliance with the ARRIVE guidelines.

## Results

### Interplay of FXN expression and lipolysis is found in adipose tissue

We first examined the *FXN* expression in adipose tissues of YG8R mice and confirmed it (Fig. S1A). A deficiency in iron-sulfur cluster biogenesis is considered the primary outcome of FXN deficiency^27^. We further confirmed that iron-sulfur cluster-related proteins were mostly significantly reduced in epididymal WAT (eWAT) and inguinal WAT (iWAT) of YG8R mice (Fig. S1B). Complex I and II of the respiratory chain are the most abundant iron-sulfur cluster-containing enzymes in mitochondria. The activities of these enzymes in eWAT and iWAT of YG8R mice were reduced due to insufficient biogenesis of iron-sulfur clusters (Fig. S1C,D). In addition, ATP contents in eWAT and iWAT of YG8R mice were also significantly reduced (Fig. S1E). Thus, these FXN-deficient mice were suitable to be used as a FRDA disease model.

**Fig. 1:**
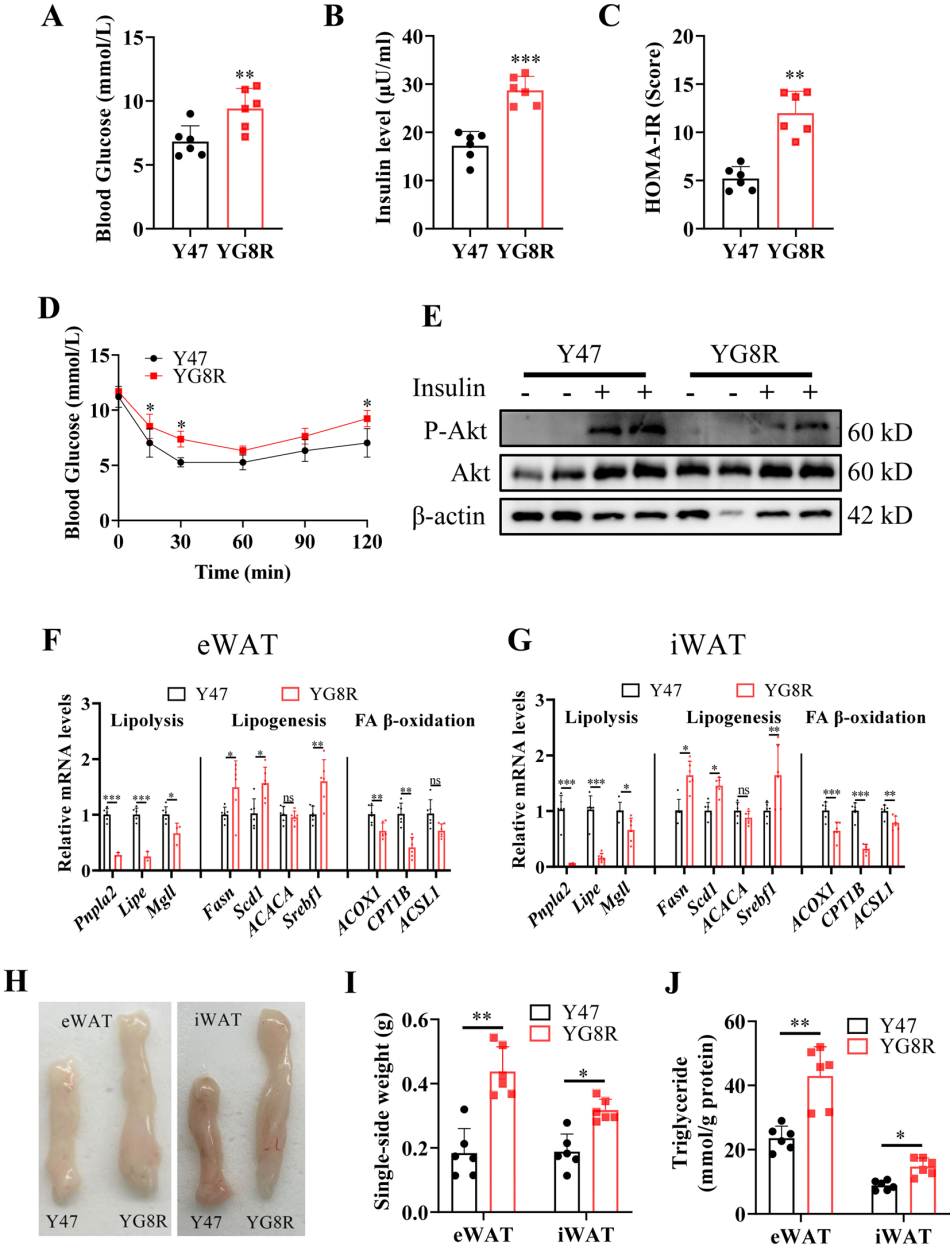
Lipolysis is most affected in adipose tissue of FXN-deficient mice YG8R, associated with insulin resistance. (**A**–**H**) Y47 and YG8R male mice at 40 weeks old, fed with a standard chow diet (n = 6). (**A**,**B**) Fasting plasma glucose (**A**) and fasting insulin levels (**B**). (**C**) Calculated HOME-IR values based on fasting plasma glucose and fasting insulin levels. (**D**) Insulin tolerance tests (ITT) to show the levels of blood glucose following insulin administration. (**E**) The total and phosphorylated Akt (s473). Five minutes after intraperitoneal insulin injection, mice were sacrificed to harvest eWAT and examined by western blot. (**F**,**G**) Expression of genes related to lipolysis, lipogenesis, and fatty-acid β-oxidation in eWAT (**F**) and iWAT (**G**). (**H**,**I**) Representative WAT and their weights of the fat pads. (**J**) TAG contents in eWAT and iWAT. *iWAT* inguinal white adipose tissue, *eWAT* epididymal white adipose tissue, *BAT* brown adipose tissue. Values are shown as mean ± SD. *t*-test was used for significance. *P < 0.05, **P < 0.01, ***P < 0.001.

As reported previously^28^, impaired insulin resistance was verified in YG8R mice at a later age (40 weeks), compared with Y47 mice (Fig. 1A–D). Since the mice gained significant weight (^28^ and Fig. S2A), the insulin resistance was considered resulting from the problem in adipose tissue, which was supported by the lessened phosphorylated protein kinase B (PKB/Akt, p-Akt, s473) in eWAT (Fig. 1E). Then, the expression of lipogenesis, lipolysis, and fatty acid (FA) β-oxidation-related genes was examined and found the entire lipid metabolism remodeled, as lipolysis and FA β-oxidation were suppressed and lipogenesis was enhanced by FXN deficiency at resting status (Fig. 1F,G). Among these crucial processes in adipose tissues, lipolysis was the most affected, particularly in the first and second steps of TAG hydrolysis by ATGL and HSL, respectively (Fig. 1F,G), which are encoded by two PPARγ-targeting genes, *Pnpla2* and *Lipe*^29,30^. Consistently, the adipose tissues (iWAT and eWAT) were grossly larger and heavier in YG8R than in Y47 mice (Fig. 1H,I). Moreover, TAG contents were increased in these tissues as well (Fig. 1J). Therefore, FXN deficiency-induced adipocyte hypertrophic expansion was speculated mainly due to the lessened lipolysis in the white adipose tissues, in agreement with previous observation^18^.

**Fig. 2:**
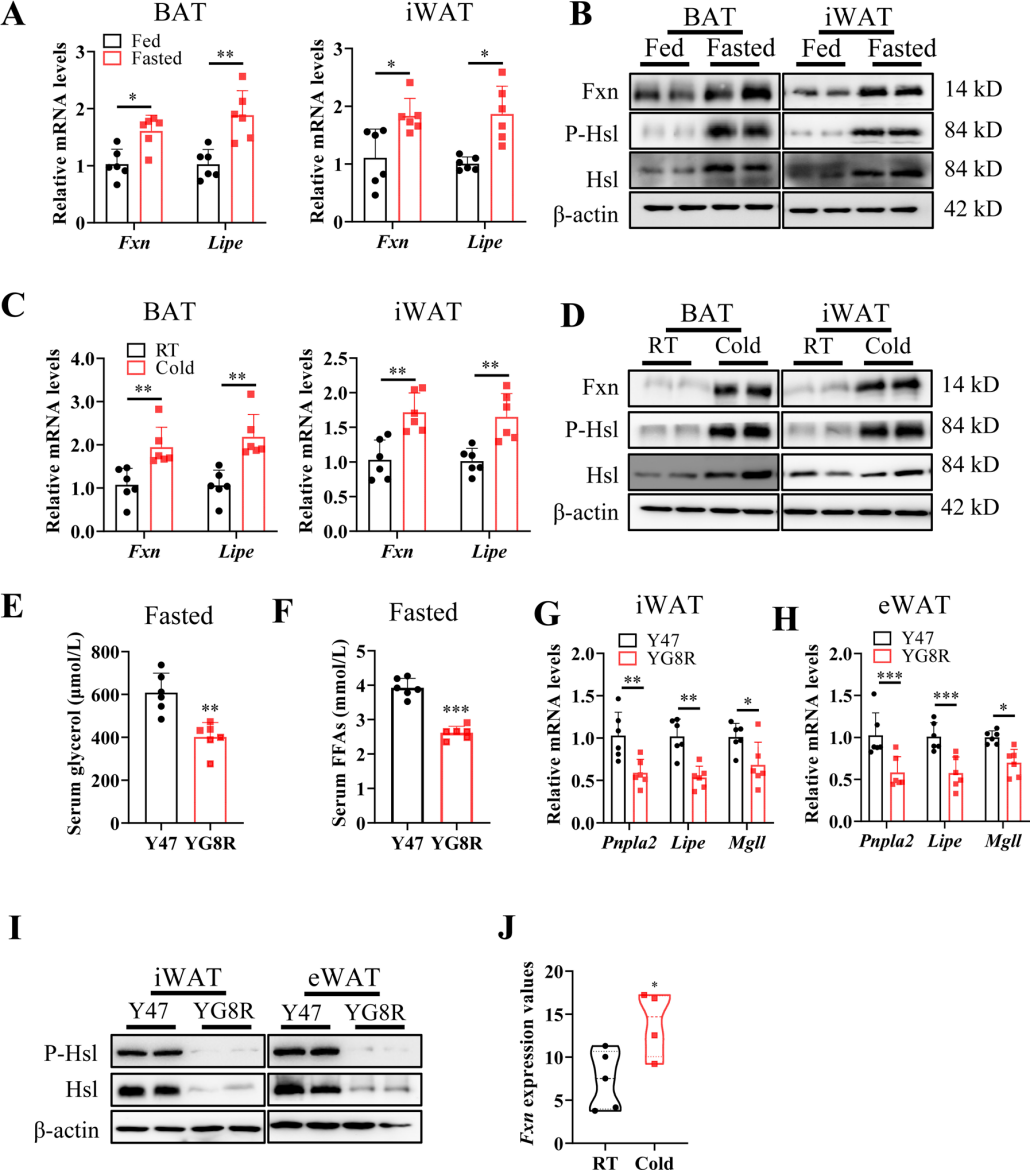
FXN plays a critical role in adipocyte lipolysis. (**A**,**B**) FXN and Hsl expression in 8-week-old wild-type male mice subjected to 24 h for fasting or normal chow feeding (n = 6). The mRNA (**A**) and protein (**B**) levels of Fxn, p-Hsl, and total Hsl in BAT and iWAT. Data were normalized to the fed group. (**C**,**D**) Fxn and Hsl expression in 8-week-old wild-type male mice subjected to cold exposure (4 °C) for 6 h (n = 6). The mRNA (**C**) and protein (**D**) levels of Fxn, p-Hsl, and Hsl in BAT and iWAT. (**E**–**I**) Y47 and YG8R male mice at 40 weeks old were fasted for 24 h (n = 6). (**E**,**F**) Serum glycerol and FFAs. (**G**,**H**) The mRNA levels of genes related to lipolysis in iWAT and eWAT of the 24-h fasted mice. Data were normalized to the Y47 mice group. (**I**) The protein levels of Hsl in iWAT and eWAT after 24-h fasting were detected by western blot analysis. (**J**) Reanalysis of publicly available RNA-seq data^33^ showing mRNA levels of Fxn in iWAT of mice following cold exposure (4 °C versus 22 °C, n = 4–5) for 3 d. *FFA* free fatty acids. Values are shown as mean ± SD. *t*-test was used for significance. *P < 0.05, **P < 0.01, ***P < 0.001.

Energy-consuming demand during food deprivation^31^ or cold exposure^32^ represents the primary driver of lipolysis. To verify the role of Fxn in adipocyte lipolysis, we either fasted or exposed the C57BL/6 strain wild-type (WT), Y47, and YG8R mice to the cold (4 °C) to stimulate adipocyte lipolysis. The expression of Fxn was significantly increased in brown adipose tissue (BAT) and WAT after 24 h of fasting at mRNA and protein levels in WT (Fig. 2A,B). A similar expression pattern was found in BAT and WAT after cold exposure (Fig. 2C,D), indicating that lipolysis strongly modulated Fxn expression in adipose tissues. To test if the upregulation is essential or is just a correlation with lipolysis, we then treated YG8R mice at 40 weeks old under similar conditions. After fasting, the lipolysis capacity of the adipose tissue may be enhanced by increasing the expression of lipolytic enzymes, resulting in increased glycerol and FFAs^31^. Compared with the control mice Y47, serum glycerol and FFAs were significantly lower in YG8R than in Y47 mice after fasting (Fig. 2E,F). The mRNA levels of related genes, such as *Pnpla2*, *Lipe*, and *Mgll*, were remarkably lower in iWAT and eWAT (Fig. 2G,H). In agreement with these, the total and phosphorylated levels of Hsl were much lower in iWAT and eWAT of YG8R mice under fasting conditions (Fig. 2I). The very similar results were obtained to fasting induction after cold exposure (Fig. S2B–F). Subsequently, we analyzed a publicly available RNA-seq dataset^33^ of iWAT from mice following cold exposure (4 °C versus 22 °C) for 3 d. We found that *Fxn* expression significantly increased in iWAT (Fig. 2J). We observed the tight correlation between Fxn expression and protein kinase A-mediated lipolysis.

### FXN deficiency induces inflammation and oxidative stress levels in adipose tissue

**Fig. 3:**
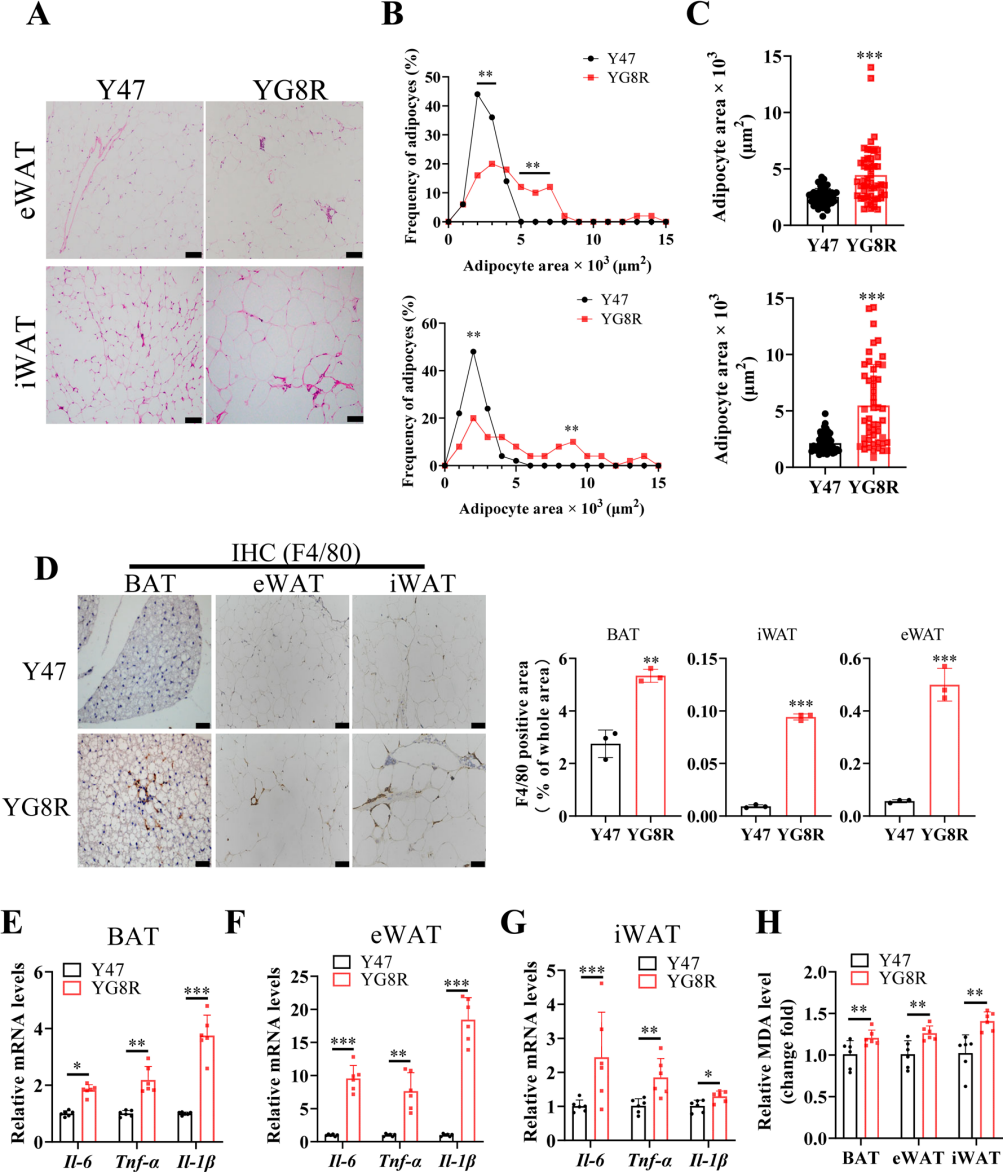
FXN deficiency induces adipocyte hypertrophic expansion and increases inflammation in WAT. (**A**–**H**) Y47 and YG8R male mice at 40 weeks old were fed a normal chow (n = 6). (**A**) Representative H&E stained eWAT and iWAT. Scale bars, 50 μm. (**B**,**C**) Size distribution patterns of eWAT and iWAT adipocytes and quantification of adipocyte sizes. (**D**) Representative images for F4/80 immunohistochemistry (IHC) staining on BAT, eWAT, and iWAT and quantification on the right. Scale bar, 20 μm. (**E**–**G**) Expression of pro-inflammatory genes in BAT (**E**), eWAT (**F**), and iWAT (**G**). (**H**) Relative fold change of MDA in BAT, eWAT, and iWAT. *TAG* triglyceride. *MDA* malondialdehyde. Data were normalized to the Y47 mice group. Values are shown as mean ± SD. *t*-test was used for significance. *P < 0.05, **P < 0.01, ***P < 0.001.

Adipose tissue hypertrophy in YG8R mice has been observed and adipocyte lipolysis is suppressed by FXN deficiency at a late age (^28^ and this study). The expansion of adipose depots can be driven by the increase in adipocyte size (hypertrophy) or the formation of new adipocytes^34^. We found a considerable proportion of larger adipocytes in YG8R than in Y47 mice (Fig. 3A–C), suggesting the driving force from hypertrophy.

The increased mechanical and hypoxic stresses of hypertrophic adipocytes contribute to adipose tissue inflammation^35^ and oxidative stress^36^. We observed an increased number of F4/80-positive cells exhibiting an inflammatory phenotype, revealed by immunohistochemistry staining (Fig. 3D). The changes were more remarkable in WAT between Y47 and YG8R than in BAT between Y47 and YG8R. In agreement with this, the expression of inflammatory factors, including *Il-6, Tnf-α,* and *Il-1β*, was found to increase in BAT, iWAT, and eWAT tissues, among which eWAT was the most affected adipose tissue in YG8R mice (Fig. 3E–G), also consistent with the very recent finding in visceral adipose tissue^37^. Malondialdehyde (MDA) is highly produced during lipid peroxidation and is commonly used as a measure of oxidative stress. MDA content increased in adipose tissue of YG8R mice (Fig. 3H). However, the serum levels of two typical inflammatory markers, Il-18 and Il-6, did not rise in YG8R mice (Fig. S3A,B). Thus, FXN deficiency-induced inflammatory cell infiltration and oxidative stress are not systemic but local, drastically, in WAT tissues. Therefore, we focused on the WAT tissue in the following experiments.

### YG8R mice exhibit a decreased lipolysis capacity in WAT at a young age far earlier than systemic abnormal hyperglycemia

**Fig. 4:**
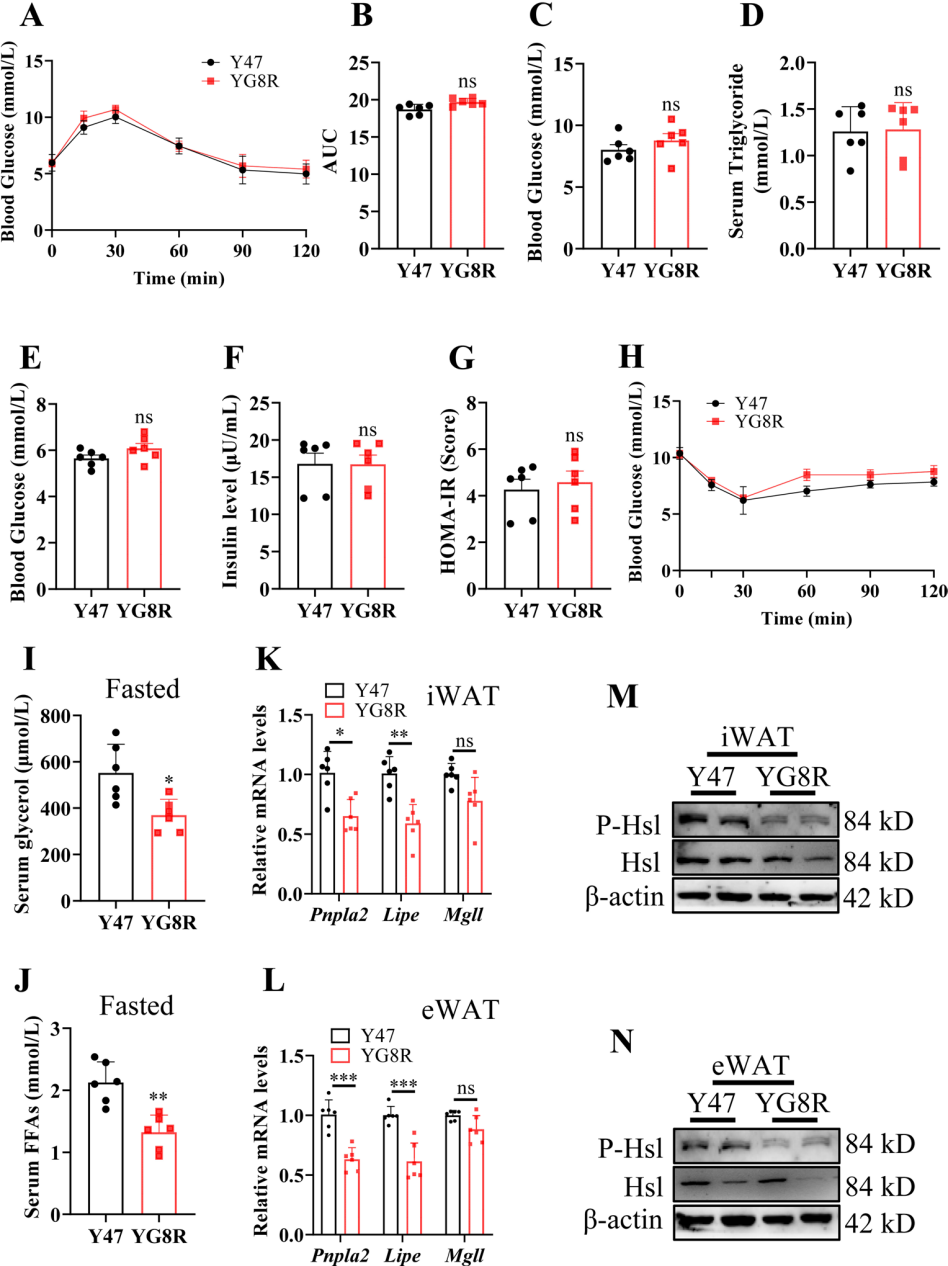
YG8R mice exhibit a decrease in lipolysis capacity in adipose tissues rather than systemic abnormal glucose metabolism at a young age. (**A**–**N**) Y47 and YG8R male mice at 16 weeks old were fed normal chow since birth. Then they were euthanized after 24-h fasting (n = 6). The samples of blood and adipose tissue were collected. (**A**) Glucose tolerance tests (GTT) to show the blood glucose following a-bolus-of-glucose administration, performed in mice before euthanizing. (**B**) Area of the under curve (AUC) calculation by analysis of the GTT data in (**A**) with subtraction of basal glucose contents. (**C**) Postprandial blood glucose levels in mice. (**D**) Serum TAG content. (**E**) Fasting plasma glucose. (**F**) Fasting insulin levels. (**G**) Calculated HOME-IR values based on fasting plasma glucose and fasting insulin levels in mice. (**H**) ITT to show the levels of blood glucose following insulin administration. (**I**,**J**) Serum glycerol and FFAs. (**K**,**L**) Expression of genes related to lipolysis in iWAT and eWAT of Y47 and YG8R mice, respectively. (**M**,**N**) Total and phosphorylated Hsl levels in iWAT and eWAT, revealed by western blot analysis. Data were normalized to the Y47 mice. Values are shown as mean ± SD. *t*-test was used for significance. *P < 0.05, **P < 0.01, ***P < 0.001.

Based on the previous experimental results, we have proven that YG8R mice exhibited abnormal glucose metabolism in older age (40 weeks), correlated with lipid dysbolism. To date, no reports on glucose and lipid metabolism are impaired in the early stage of FRDA patients and mice. We here showed that YG8R mice did not develop diabetes manifestation until 16 weeks old (referred to as “young”), exhibiting similar glucose intolerance, fasting and postprandial blood glucose, and serum TAG content in young YG8R to Y47 mice (Fig. 4A–F). Furthermore, YG8R mice exhibited very mild, without significance, changes in insulin sensitivity compared with Y47 mice at 16 weeks of age (Fig. 4G,H), suggesting a late diabetes manifestation under FXN-deficient conditions.

However, serum glycerol and FFAs were significantly less in YG8R mice after fasting, compared to the Y47 mice (Fig. 4I,J). The mRNA levels of *Pnpla2* and *Lipe,* not *Mgll*, were significantly decreased in iWAT and eWAT of YG8R mice (Fig. 4K,L). In addition, similar to the elder YG8R mice, the expression of lipogenesis-related genes in iWAT and eWAT was increased, and the expression of FA β-oxidation-related genes was decreased compared with Y47 mice (Fig. S4). Moreover, the protein and phosphorylation levels of Hsl were also significantly reduced (Fig. 4M,N), indicating that YG8R mice at a young age exhibited a weakened lipolysis capacity in WAT tissues before the appearance of abnormal glucose metabolism.

### Enhanced lipolysis compensates for insulin insensitivity resulting from FXN deficiency in adipocytes

**Fig. 5:**
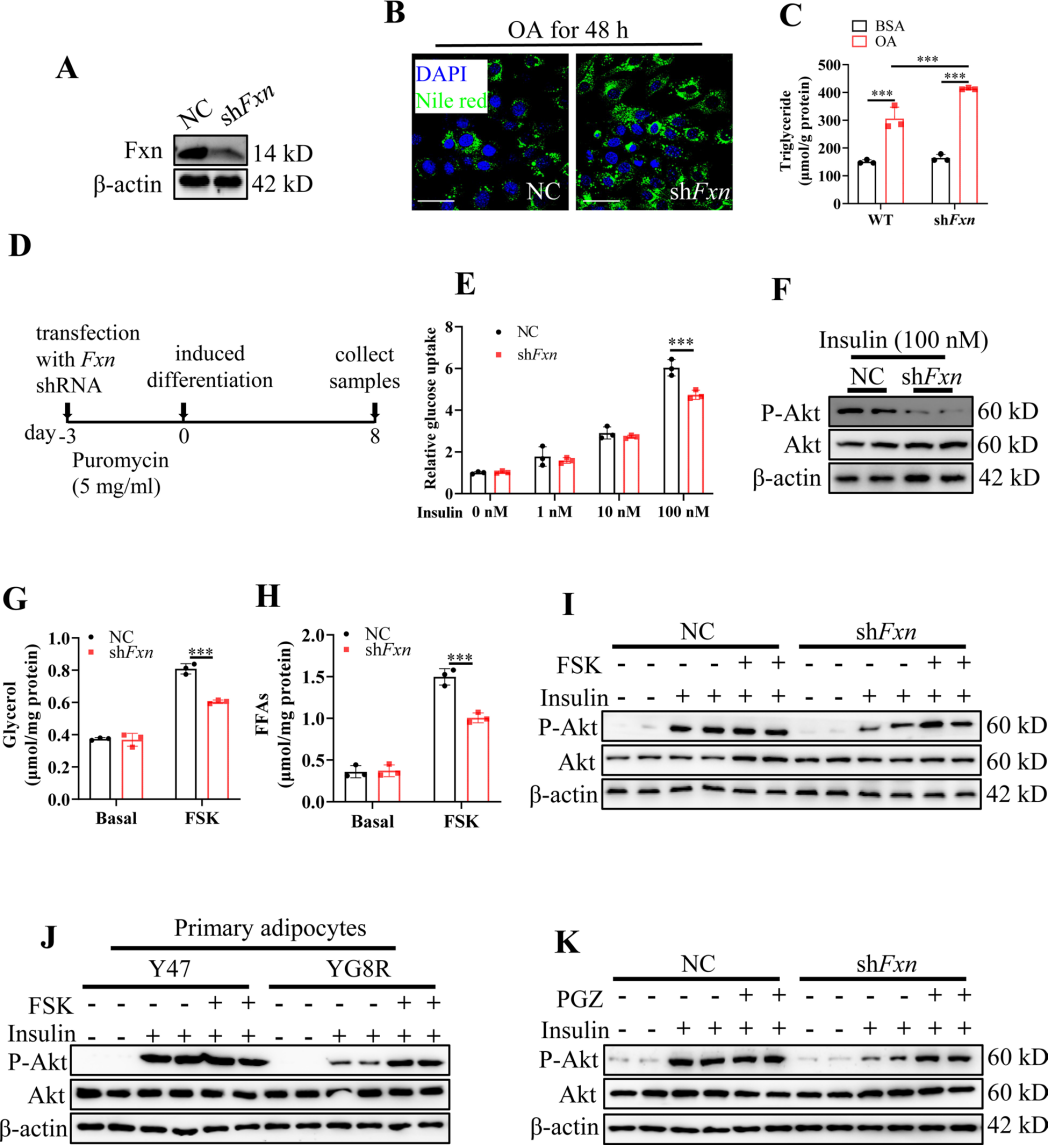
FXN deficiency impairs insulin sensitivity in adipocytes. (**A**) Fxn knockdown efficiency in 3T3-L1 cells using Fxn shRNA to generate a stable cell line. Samples were used for subsequent experiments. (**B**) 3T3-L1 preadipocytes were stained with Nile red for lipid droplets and visualized by confocal microscope. The stable cell lines of Fxn-deficiency (sh*Fxn*) and control (NC) were treated with 300 μM of oleic acid (OA) for 48 h, followed by Nile staining. Scale bars, 50 μm. (**C**) TAG content in 3T3-L1 preadipocytes after OA treatment (300 μM). The same sampling was used as in (**B**). (**D**) A schematic diagram of experimental design for sh*Fxn* 3T3-L1 differentiation. (**E**) Relative glucose uptake levels of NC and sh*Fxn* 3T3-L1 cells following insulin treatment for 20 min with indicated concentration. (**F**) The protein levels of p-Akt (s473) and Akt in response to insulin (100 nM) addition in differentiated NC and sh*Fxn* 3T3-L1 cells. (**G**,**H**) The contents of glycerol and FFAs released into the medium after cells were treated with or without FSK (50 μM) for 4 h. (**I**–**K**) The responses of FXN deficient cells to lipolysis agonist FSK (**I**,**J**, 20 μM for 48 h) or PGZ (**K**, 10 μM for 48 h) and insulin addition (100 nM, 20 min). Differentiated NC and sh*Fxn* 3T3-L1 cells for (**I**) and (**K**); Differentiated primary adipocytes from Y47 and YG8R mice for (**J**). *NC* negative control. Values are shown as mean ± SD. n = 3 for each. *t*-test was used for significance. *P < 0.05, **P < 0.01, ***P < 0.001.

To further investigate that the effect of FXN on adipocyte lipolysis affected insulin sensitivity, we used 3T3-L1 cells based on their potential to differentiate from fibroblasts to adipocytes. We first knocked down *Fxn* by shRNA (sh*Fxn* cells) (Fig. 5A). Oleic acid (OA) may stimulate lipid accumulation in 3T3-L1 preadipocytes and induce lipid droplet enlargement^38^. Then, we used OA to challenge the sh*Fxn* cells, and the lipid droplet sizes were determined by Nile staining. The results showed that Fxn deficiency increased lipid droplet sizes and promoted lipid droplet abnormal growth and accumulation, whereas more tiny lipid droplets distributed in the presence of OA in the control cells (Fig. 5B). Meanwhile, cellular TAG was increased in *Fxn*-deficient preadipocytes after OA treatment attributing to the reduced TAG hydrolysis (Fig. 5C), indicating a well-established *Fxn*-deficient adipocyte model.

Subsequently, we analyzed cellular glucose uptake using a 2-NBDG Glucose Uptake Assay Kit. The results showed that the glucose uptake was significantly less in the sh*Fxn* differential cells than that of the NC cells at a concentration 100 nM of insulin for 20 min (Fig. 5D,E). Then, the optimized concentration was used. We found that protein levels of p-Akt/Akt dramatically decreased in sh*Fxn* cells in response to insulin addition (Fig. 5F). To evaluate the effect of Fxn deficiency on the lipolysis capacity of adipocytes, the differentiated NC and sh*Fxn* 3T3-L1 cells were treated with forskolin (FSK, 50 μM), an agonist of lipolysis^39^, for 4 h. Consistent with in vivo results, the levels of FFAs and glycerol in the culture media of sh*Fxn* cells were significantly lower than in the NC group (Fig. 5G,H). These data indicated that FXN deficiency in adipocytes reduced insulin sensitivity and lipolysis capacity.

Since the aberrant lipolysis is induced before insulin resistance in YG8R mice, we wonder if enhanced lipolysis could improve insulin sensitivity. Then, we used FSK to stimulate the lipolysis. The results showed that phosphorylation of Akt was further boosted by FSK treatment following insulin treatment in sh*Fxn* 3T3-L1 cells (Fig. 5I). A similar effect was observed with primary adipocytes derived from YG8R mice (Fig. 5J). Pioglitazone (PGZ), an agonist of PPARγ, also increased the phosphorylation of Akt following insulin treatment in sh*Fxn* 3T3-L1 cells (Fig. 5K). Taken together, the results indicate that lipolysis enhancement can compensate for insulin insensitivity resulting from FXN deficiency.

### FXN deficiency aggravates HFD-induced obesity, diabetes, and liver steatosis at a young age

**Fig. 6:**
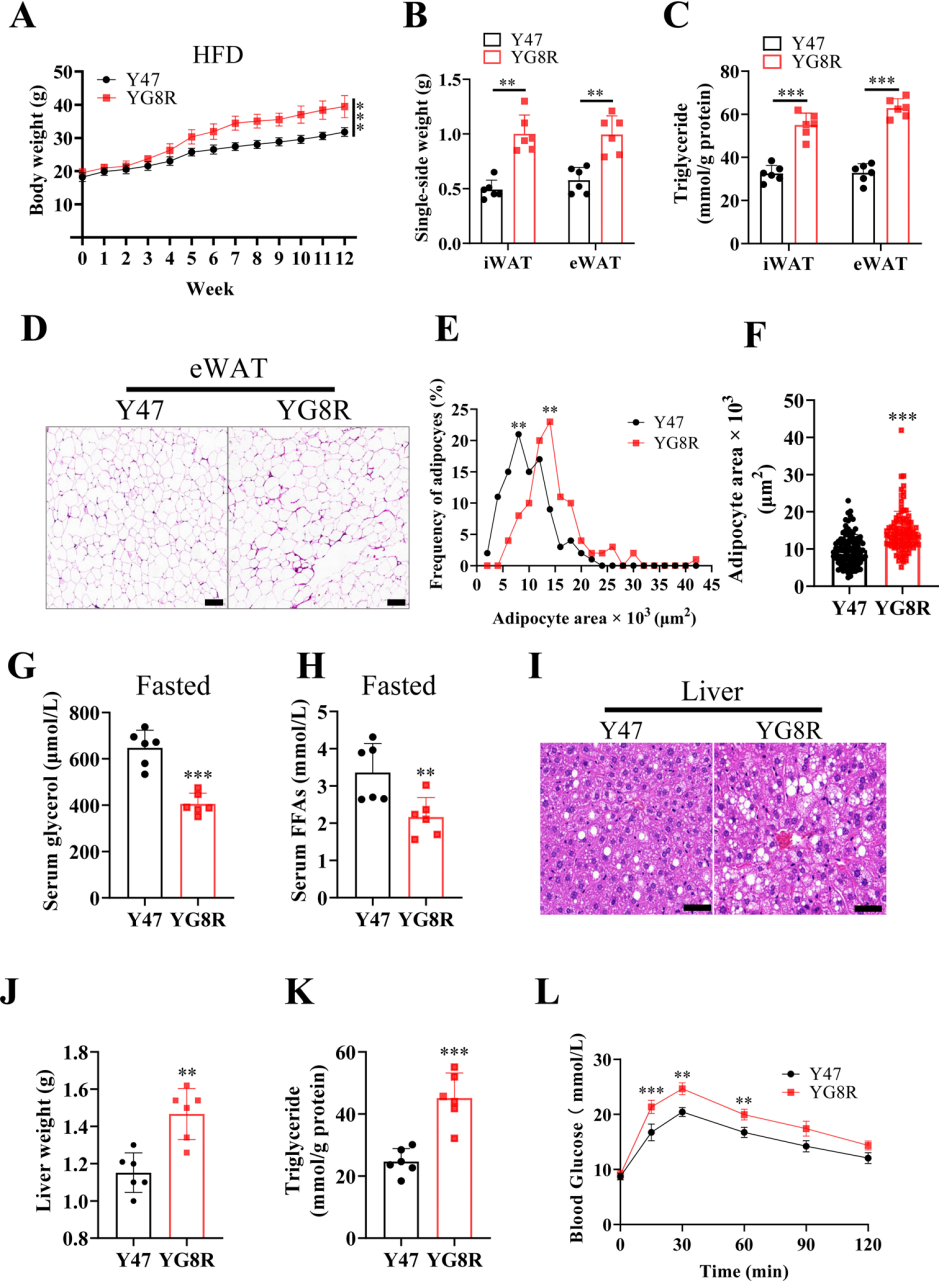
FXN deficiency aggravates diet-induced obesity, insulin resistance, and liver steatosis. (**A**–**L**), Y47 and YG8R male mice at 8 weeks old were fed with high fat diet (HFD) for 12 weeks (n = 6). The mice were euthanized after fasting for 18 h. (**A**) Body weights. (**B**) Weights of fat pads. (**C**) TAG contents in eWAT and iWAT. (**D**) Representative H&E stained eWAT. Scale bars, 200 μm. (**E**,**F**) Proportion distribution and sizes of adipocytes. (**G**,**H**) Serum glycerol and FFAs. (**I**) Representative H&E-stained liver to show the steatosis. Scale bars, 50 μm. (**J**) Weights of liver. (**K**) TAG contents in the liver. (**L**) GTT to show the levels of blood glucose following a-bolus-of-glucose administration. Data were normalized to the Y47 mice. Values are shown as mean ± SD. *t*-test was used for significance. *P < 0.05, **P < 0.01, ***P < 0.001.

The role of FXN in lipid metabolism was further examined in vivo. Y47 and YG8R mice were fed an HFD at 8 weeks for 12 weeks. After HFD treatment, YG8R mice gained more body weight (Fig. 6A) and significantly higher adipose-tissue weights than Y47 mice (Fig. 6B). YG8R mice also accumulated more TAGs in adipose tissues (Fig. 6C). Consistently, the sizes of adipocytes in eWAT of YG8R mice are markedly larger than in Y47 mice and the proportion of larger adipocytes significantly increased (Fig. 6D–F). After fasting for 18 h, serum glycerol and FFAs were markedly less in YG8R than in Y47 mice (Fig. 6G,H).

Liver is another crucial tissue for lipid metabolism. Interestingly, no pathological changes were observed in the liver of YG8R mice with standard chow (Fig. S5A–C). However, after 12 weeks of HFD, histologic examination demonstrated chronic liver disorder with macrovesicular steatosis and ballooning degeneration in the liver of YG8R mice (Fig. 6I). Consistently, the liver was heavier (Fig. 6J), and its TAG content was prominently more in YG8R than in Y47 (Fig. 6K). In agreement with this, serum ALT and AST were increased in YG8R mice (Fig. S5D,E). As expected, YG8R mice showed more severe glucose intolerance than Y47 mice after fasting (Fig. 6L), indicating that FXN deficiency aggravated lipid-metabolic disorders caused by excessive nutrition.

### PGZ treatment improves glucose metabolism in YG8R mice by enhancing adipocyte lipolysis capacity

**Fig. 7:**
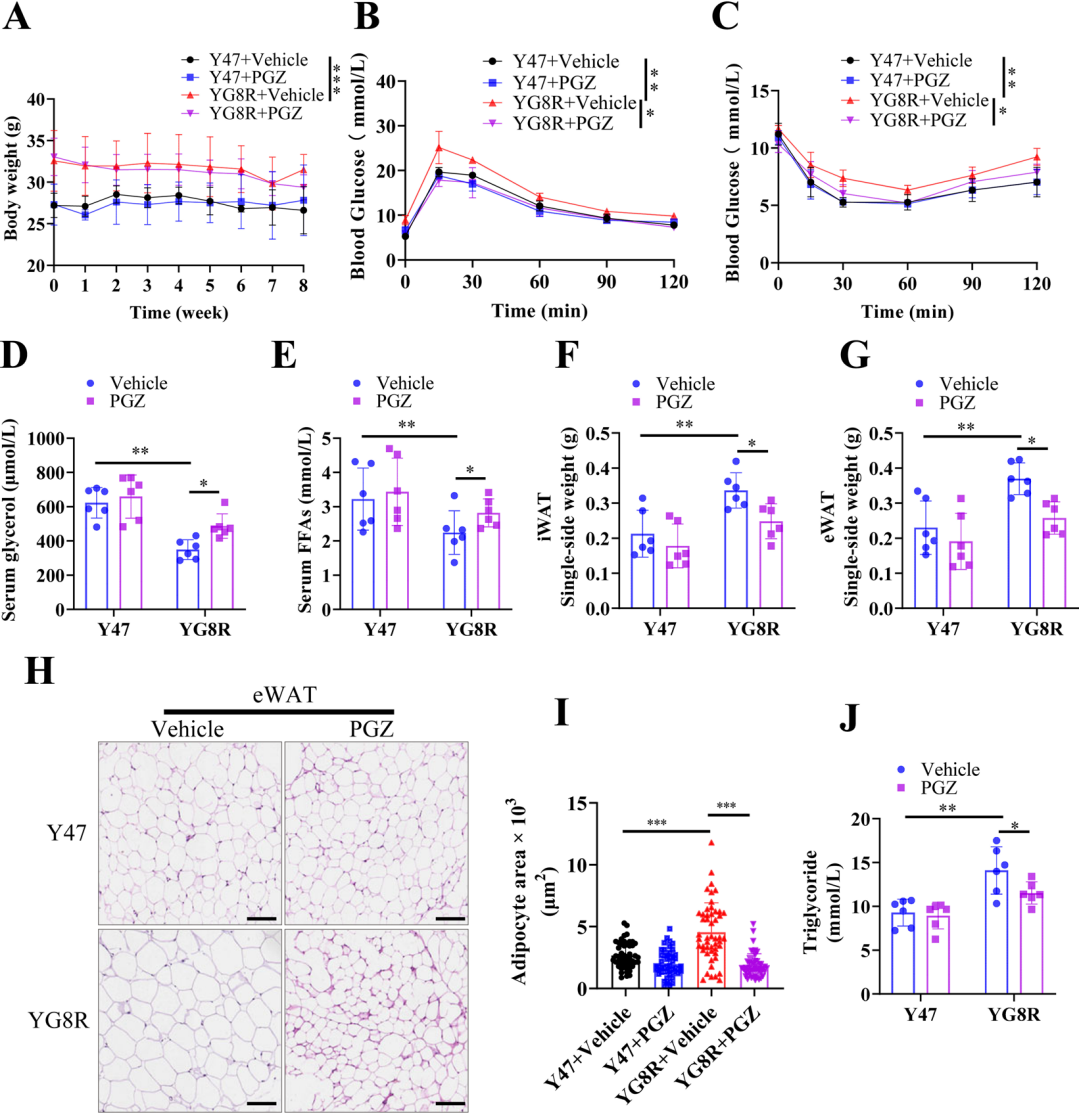
PGZ treatment improves glucose metabolism in YG8R mice by enhancing adipose tissue lipolysis capacity. (**A**–**J**) Y47 and YG8R mice at 32 weeks old were treated with PGZ (25 mg/kg/d; oral administration by gavage) for 8 weeks (n = 6). (**A**) Body weights. (**B**) GTT to show the levels of blood glucose following a-bolus-of-glucose administration. (**C**) ITT to show the levels of blood glucose following insulin administration. (**D**,**E**) Serum glycerol and FFAs. (**F**,**G**) Weights of iWAT and eWAT. (**H**) Representative H&E stained eWAT. Scale bars, 100 μm. (**I**) Quantification of adipocyte sizes. (**J**) TAG contents in eWAT. *PGZ* Pioglitazone. Values are shown as mean ± SD. *t*-test was used for significance. *P < 0.05, **P < 0.01, ***P < 0.001.

Fasting as a PPARγ agonist may potentiate lipolysis, and PGZ, clinically as a PPARγ agonist, is prescribed to T2D patients in combination with metformin. YG8R mice benefited from PGZ administration to prevent neurological disorders^16^. We expected the alleviation from the aberrant lipolysis if YG8R mice were treated with PGZ. The results showed that PGZ treatment (25 mg/kg/d; oral administration by gavage) for 8 weeks at 8 months of age did not affect mice weight (Fig. 7A). However, glucose intolerance was significantly improved (Fig. 7B), associated with an increase in insulin sensitivity in YG8R mice (Fig. 7C). After fasting, serum glycerol and FFAs increased in YG8R mice by PGZ treatment (Fig. 7D,E). Interestingly, the weights of iWAT and eWAT were significantly decreased in YG8R mice by PGZ treatment (Fig. 7F,G). However, the body weight was kept constant (Fig. 7A). The fat weight of Y47 mice also slightly decreased by PGZ treatment without significance. PGZ treatment prevented adipocyte expansion (Fig. 7H) and reduced the sizes of adipocytes in YG8R mice (Fig. 7I). Meanwhile, TAG content was decreased in adipose tissue of YG8R mice (Fig. 7J). Consistent with previous studies^40^, we found PGZ treatment significantly increased the protein expression of FXN in the eWAT of YG8 mice (Fig. S6). Together, PGZ treatment improved glucose metabolism in YG8R mice by enhancing adipocyte lipolysis.

## Discussion

Abnormal glycemic control, increased triglyceride levels, and T2D are more frequent in FRDA patients than in the general population and concur with the severity of FRDA^3^. These phenotypes were recapitulated in *FXN* deficient YG8R mice at elder age, associated with insulin resistance and lipolysis retardation. Interestingly, we found that FXN expression correlated well with PPARγ-mediated lipolysis-related genes under static and lipolysis-induction conditions from a young age of YG8R mice. *FXN* deficiency primarily impairs WAT lipolysis from the young age, far earlier than insulin-resistance manifestation, and aggravates diet-induced diabetes and insulin resistance. Enhancement of lipolysis upregulates FXN expression and significantly improves insulin sensitivity. Our results revealed the interaction of FXN and PPARγ signaling pathway, shedding light on a pharmaceutical strategy for a positive feedback development in treating FRDA, beneficial from both diabetes and neurodegeneration.

The expression of mitochondrial protein FXN remarkably increased following fasting or cold exposure and correlated well with the increase of the HSL phosphorylation in adipose tissue. On the other hand, experiments with YG8R mice clearly answered that FXN deficiency reduced the PPARγ-mediated ATGL and HSL expression. This interaction indicates the crucial role of mitochondrial metabolism in lipolysis. In adipose tissue, ATGL and HSL are responsible for more than 90% of TAG hydrolysis^41^. Thus, FXN deficiency causes the lipid droplet deposit, enlarges the adipocytes, and increases the WAT weight in YG8R mice, consistent with recent studies^15,18,37,42^. Notably, lipid dysbolism is an early event, supported by other observations^43^, before glycemia symptoms, indicating that T2D manifestation, at least partially, results from impaired lipolysis capacity in adipose tissue in FRDA patients.

When the intracellular lipid concentration exceeds physiological levels, it exerts lipotoxicity, involving lipid peroxidation. Lipid peroxidation was considered the major cause of cell toxicity in the FRDA mouse model^15,44^. It is well-accepted that FXN deficiency causes oxidative stress, which was found early in yeast^45^ and fruit flies^46^. Hydrogen peroxidase to scavenge H_2_O_2_^47^ or ApoD-analog expression^46^ to repress lipid peroxides effectively counteract most of the effects caused by FXN deficiency, including neurological impairment in fruit flies. In agreement with these and the recent studies^18,37^, our results fit very well with previous observation of an increase in intracellular lipid peroxides. Inflammatory cell infiltration was also found in adipose tissues in this study. Still, serum inflammatory cytokines remain unchanged, indicating the local, not systemic, inflammation in adipose tissue of *FXN*-deficient mice.

PPARγ plays essential roles in regulating fundamental cellular processes such as differentiation, development, and metabolism (carbohydrate and lipid). It is also a key regulator of mitochondrial function, biogenesis, and anti-oxidation effects^48^. Dysregulation of the PPARγ pathway has been found to play a vital role in heart and skeletal muscle by dysregulating the PPARγ coactivator *Pgc1a* and transcription factor *Srebp1* in FRDA mouse models^14^. We found that the PPARγ-targeted genes expressed significantly lower in YG8R mice than in Y47 mice. PPARγ has been demonstrated to be a main lipolytic transcription factor in differentiated adipocytes^29^. Therefore, PPARγ agonist PGZ administration significantly reduced the levels of plasma glucose and increased insulin sensitivity in our study. Previous studies have reported that both PGZ^40^ and leriglitazone^16^ as PPARγ agonists increase FXN expression to rescue cell survival and improve neuron degeneration. Combined with our study, an interplay relationship between FXN and PPARγ signaling is proposed, and the detailed mechanism is under investigation. In our study, PGZ did not change the weights of Y47 and YG8R mice, although it had been reported to increase subjects' weight in another work^49^. The benefits likely resulted from increased lipolysis and FXN expression, as we observed in this study.

Early intervention targeting lipolysis is plausible to be considered for clinical treatment. We used three ways to potentiate lipolysis directly: fasting, cold exposure, and FSK treatment. The former two dramatically increased FXN expression in mice, and the latter one increased insulin sensitivity in differential 3T3-L1 sh*FXN* cell model. Insulin-induced Akt phosphorylation was increased in sh*Fxn* cells and primary adipocytes of YG8R mice after FSK treatment. Therefore, we expected that increasing the lipolysis levels in adipocytes could improve insulin resistance caused by FXN deficiency. The intervention at a young age might maximize the benefits.

## Conclusions

In conclusion, *FXN* deficiency impairs the lipolysis, far earlier than insulin resistance. The insulin resistance is mainly due to the decrease in lipolysis in WAT. An interplay relationship between FXN and PPARγ signaling is revealed. The benefit from early intervention with lipolysis is expected in FRDA patients.

### Supplementary Information

Supplementary Information.

## Data Availability

All data generated or analysed during this study are included in this published article and its supplementary information files.

## Abbreviations

FRDA: Friedreich ataxia
FXN: Frataxin
PPARγ: Peroxisome proliferator-activated receptor gamma
ATGL: Adipose triglyceride lipase
HSL: Hormone-sensitive lipase
