# Supplementary material for: Interplay of FXN expression and lipolysis in white adipocytes plays a critical role in insulin sensitivity in Friedreich’s ataxia mouse model
Source: Sci Rep. 2024 Aug 27;14:19876. doi: 10.1038/s41598-024-71099-7 (PMC11350209; doi:10.1038/s41598-024-71099-7)
Supplement: Supplementary file 1 — Supplementary Information. [file 41598_2024_71099_MOESM1_ESM.pdf]

Interplay of FXN expression and lipolysis in white adipocytes plays a critical role in insulin sensitivity in Friedreich's ataxia mouse model

Lin Wu[1*], Fei Huang[2*], Lu Yang[1], Liu Yang[1], Zichen Sun[1], Jinghua Zhang[1], Siyu Xia[2], Hongting Zhao[1], Yibing Ding[1], Dezhi Bian[2**], Kuanyu Li[1**]

[1]Jiangsu Key Laboratory of Molecular Medicine, Medical School, Nanjing University, Nanjing, 210093 P. R. China

[2]Endocrinology Department, Yancheng First Hospital Affiliated Hospital of Nanjing University Medical School, Yancheng, 224000, P. R. China

*Lin Wu and Fei Huang contributed equally to this work.

**To whom correspondence should be addressed:

State Key Laboratory of Pharmaceutical Biotechnology, Jiangsu Key Laboratory of Molecular Medicine, Medical School, Nanjing University, Nanjing 210093, China.

Tel: +86-25-83593192;

Email: likuanyu@nju.edu.cn; bianrz2008@163.com

Table S1. Nucleic acid sequences used in the present study

Gene	Forward	Reverse
**(1) qPCR primer sequences**		
Pnpla2	GGAACCAAAGGACCTGATGACC	ACATCAGGCAGCCACTCCAACA
Lipe	GCTCATCTCCTATGACCTACGG	TCCGTGGATGTGAACAACCAGG
Mgll	GACACCATCCAGAAGGACTACC	GATTGGCAAGGACCAGAGGTGA
Fxn	CGGAGCTGGAGTAGCATGTG	CGGCGTTGACTGTGACATGTAG
Fasn	CACAGTGCTCAAAGGACATGCC	CACCAGGTGTAGTGCCTTCCTC
Scd1	GCAAGCTCTACACCTGCCTCTT	CGTGCCTTGTAAGTTCTGTGGC
ACACA	GTTCTGTTGGACAACGCCTTCAC	GGAGTCACAGAAGCAGCCCATT
Srebf1	CGACTACATCCGCTTCTTGCAG	CCTCCATAGACACATCTGTGCC
ACOX1	GCCATTCGATACAGTGCTGTGAG	CCGAGAAAGTGGAAGGCATAGG
CPT1B	ATGTATCGCCGCAAACTGGACC	CTCTGAGAGGTGCTGTAGCAAG
ACSL1	ATCAGGCTGCTTATGGACGACC	CCAACAGCCATCGCTTCAAGGA
Il-6	TGAACAACGATGATGCACTTG	CTGAAGGACTCTGGCTTTGTC
Tnf-α	ACGTCGTAGCAAACCACCAA	GCAGCCTTGTCCCTTGAAGA
Il-1β	CAGGCAGGCAGTATCACTCA	AGGCCACAGGTATTTTGTCG
18s	GGCTACCACATCCAAGGAA	GCTGGAATTACCGCGGCT
**(2) Sequences of shRNA used in the present study**		
Fxn	5’-GAGTTCTTTGAAGACCTCGCA-3’	

Table S2. Antibodies used in the present study

Antigen	Vendor	Catalog number
p-HSL	Cell Signaling Technology	45804
HSL	proteintech	17333-1-AP
p-Akt	Cell Signaling Technology	4058S
Akt	Cell Signaling Technology	9272
IscU	Abcam	ab180532
Ndufs1	proteintech	12444-1-AP
Sdhb	Abcam	ab178423
Uqcrfs1	proteintech	1843-1-AP
β-actin	Bioworld	AP0060
F4/80	Servicebio	GB113373-100

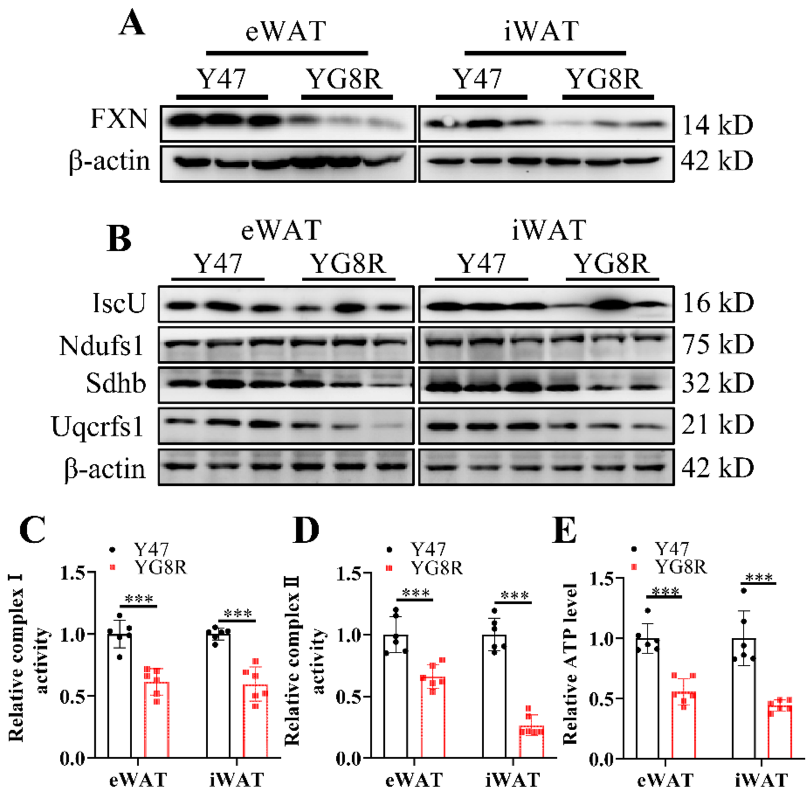

Figure S1. Frataxin (FXN) deficiency impairs respiratory chain complex activities in eWAT and iWAT. A, The protein levels of FXN in eWAT and iWAT. B, The expression of iron sulfur cluster related proteins in eWAT and iWAT. C and D, The relative activities of complexes I and II in eWAT and iWAT. E, The relative ATP levels in eWAT and iWAT. Values are shown as mean ± SD. t-test was used for significance. *P<0.05, **P < 0.01, ***P < 0.001.

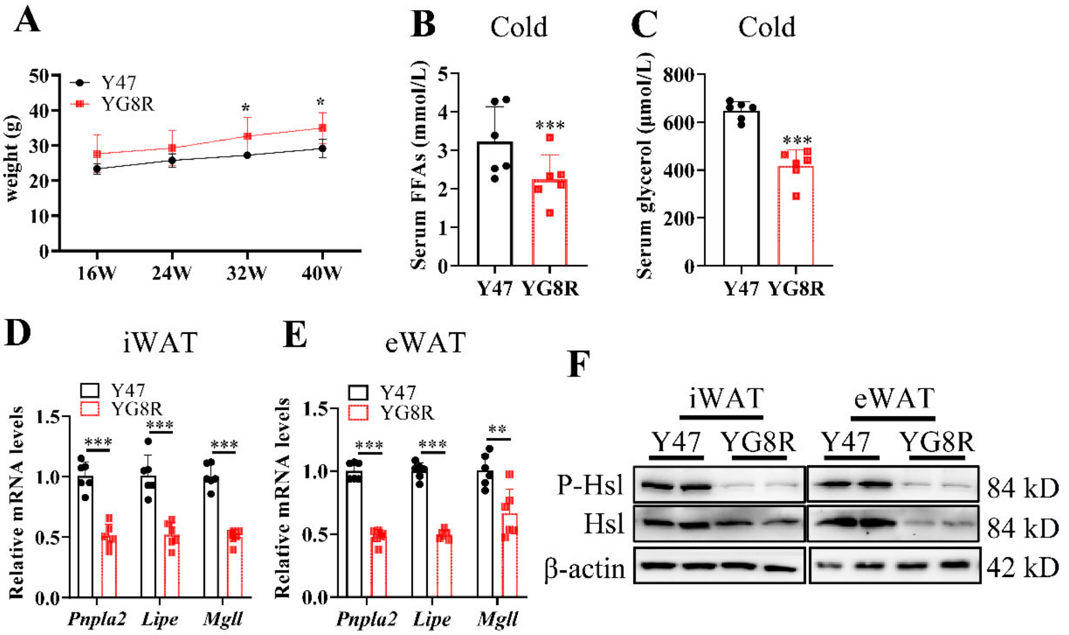

Figure S2. FXN plays a critical role in adipocyte lipolysis. A, The weights of Y47 and YG8R mice. B-F, Y47 and YG8R male mice at 40 weeks old were exposed to cold (4℃) for 6 h (n = 6). B and C, Serum FFAs (B) and glycerol (C). D and E, Expression of genes related to lipolysis in iWAT (D) and eWAT (E) of Y47 and YG8R mice after cold exposure. F, The levels of total Hsl and p-Hsl in iWAT and eWAT after cold exposure, detected by western blot analysis. Values are shown as mean ± SD. t-test was used for significance. *P<0.05, **P < 0.01, ***P < 0.001.

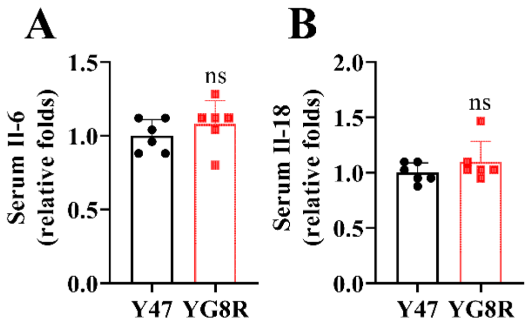

Figure S3. FXN deficiency does not cause systemic inflammation. Related to Fig. 3. A and B, Serum Il-6 and Il-18, detected by ELISA. Values are shown as mean ± SD. t-test was used for significance. n=6, *P<0.05, **P < 0.01, ***P < 0.001.

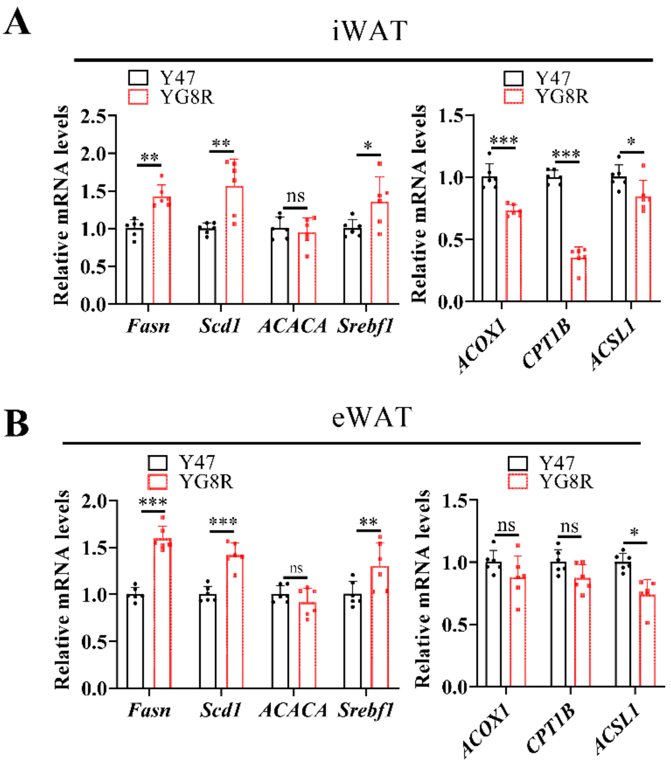

Figure S4. **Expression of genes related to lipogenesis and fatty-acid β-oxidation in iWAT (A) and eWAT (B) in 4-month-old Y47 and YG8R mice after fasting 24 h.** Values are shown as mean ± SD. t-test was used for significance. n=6 *P<0.05, **P < 0.01, ***P < 0.001.

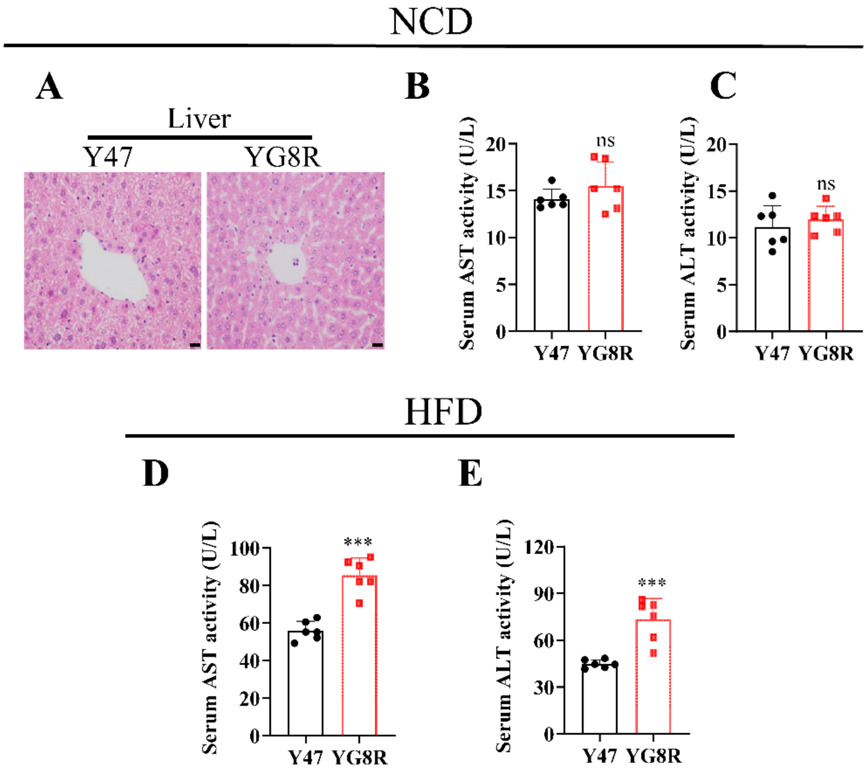

Figure S5. FXN deficiency does not cause pathological changes in the liver with normal chow at a young age. A-C, Y47 and YG8R male mice at 20 weeks, fed with NCD, were euthanized after 18-h fasting (n = 6). A, Representative H&E staining with liver sections. Scale bars, 20 μm. B and C, Serum levels of AST and ALT. D and E, Serum levels of AST and ALT of Y47 and YG8R mice with 12-week HFD. NCD: Normal chow diet. HFD: High fat diet. Values are shown as mean ± SD. t-test was used for significance. *P<0.05, **P < 0.01, ***P < 0.001.

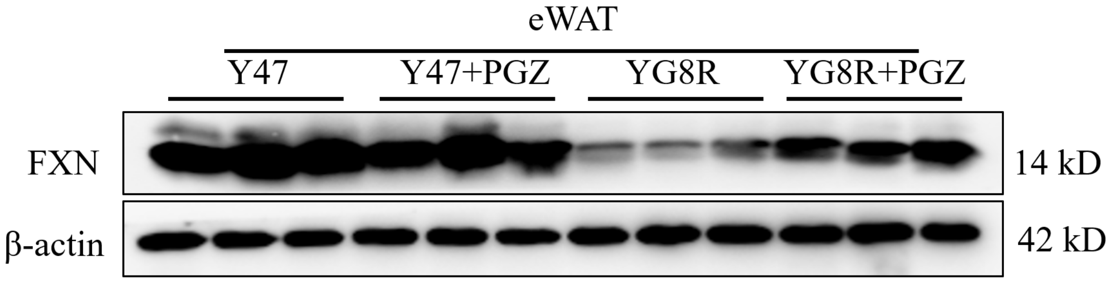

Figure S6. PGZ treatment increases the protein expression of FXN in adipose tissue of YG8R mice. The protein expression of FXN in eWAT of Y47 and YG8R mice, detected by western blot analysis.

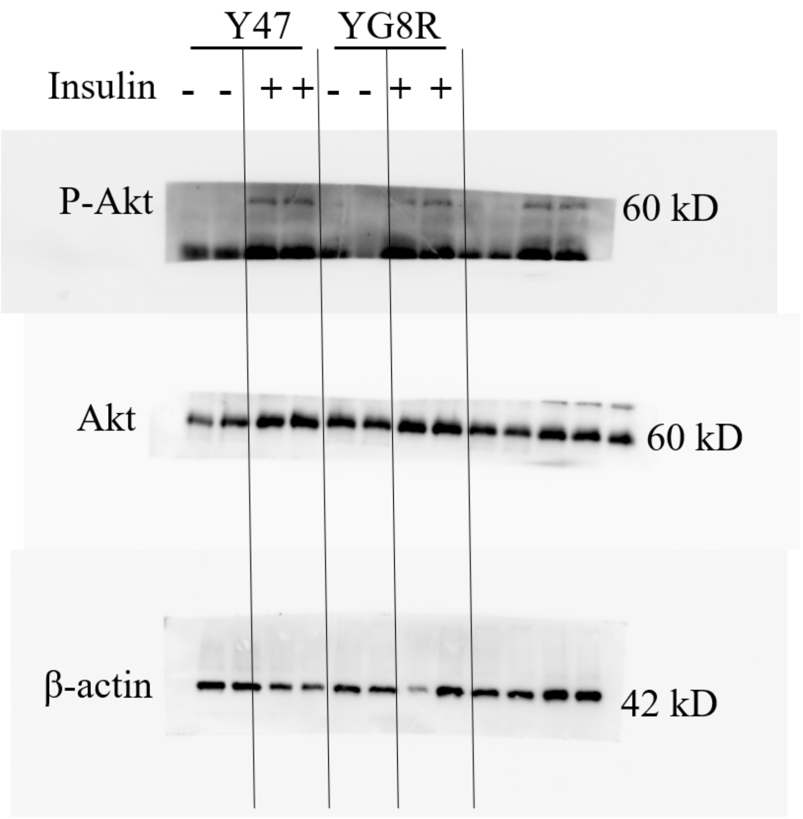

Figure S7. Uncropped images of immunoblots for figure 1E.

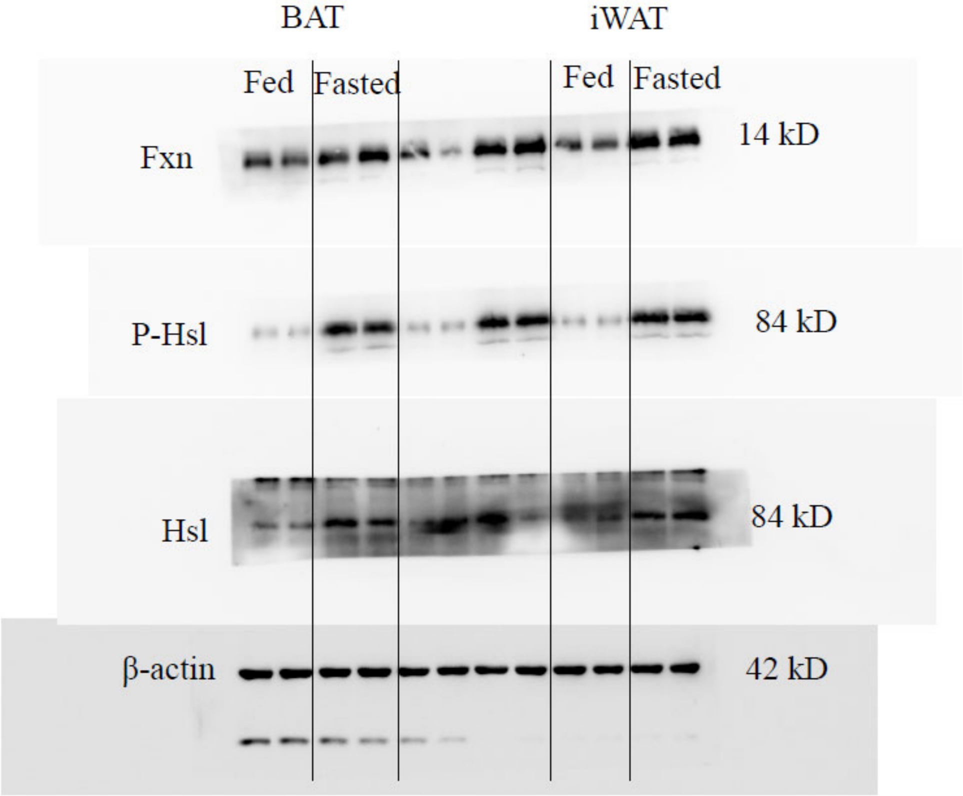

Figure S8. Uncropped images of immunoblots for figure 2B.

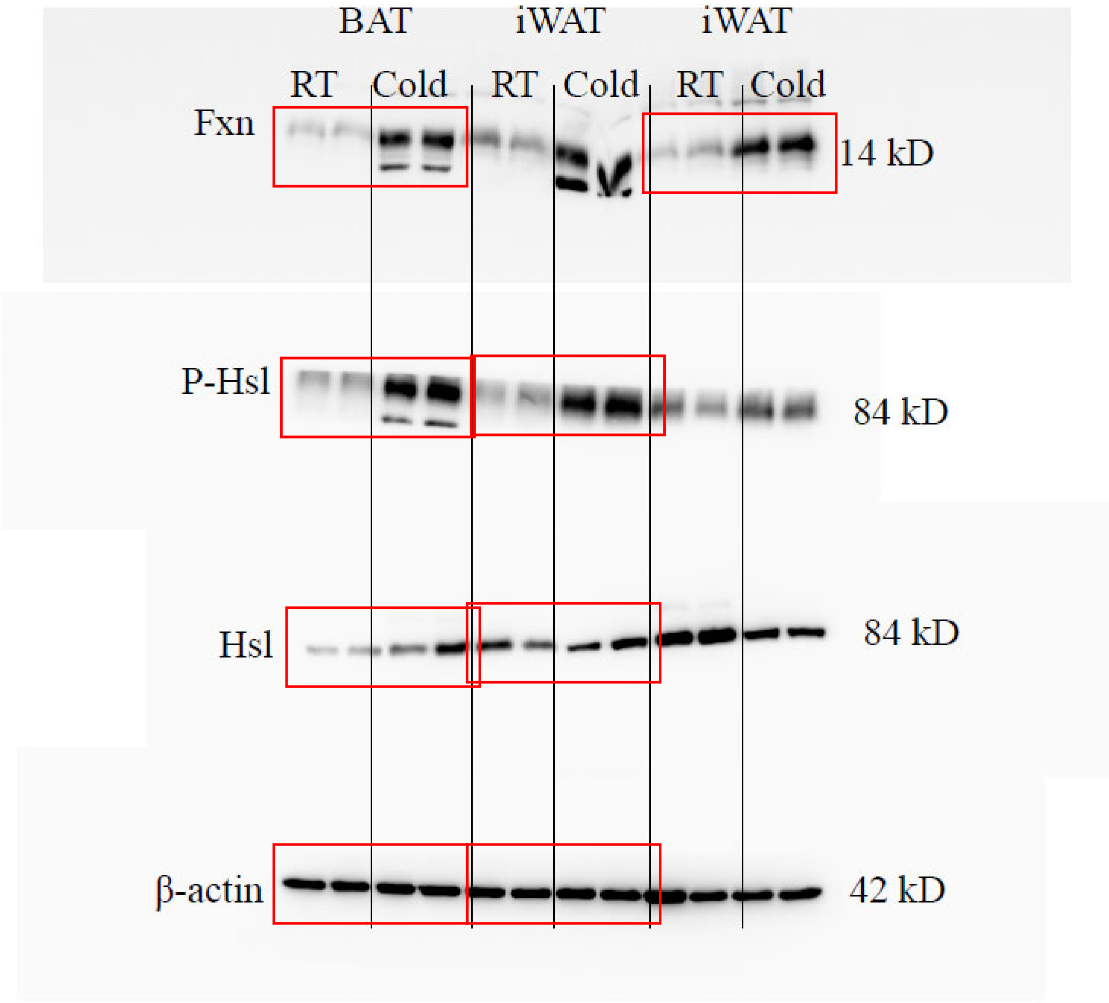

Figure S9. Uncropped images of immunoblots for figure 2D.

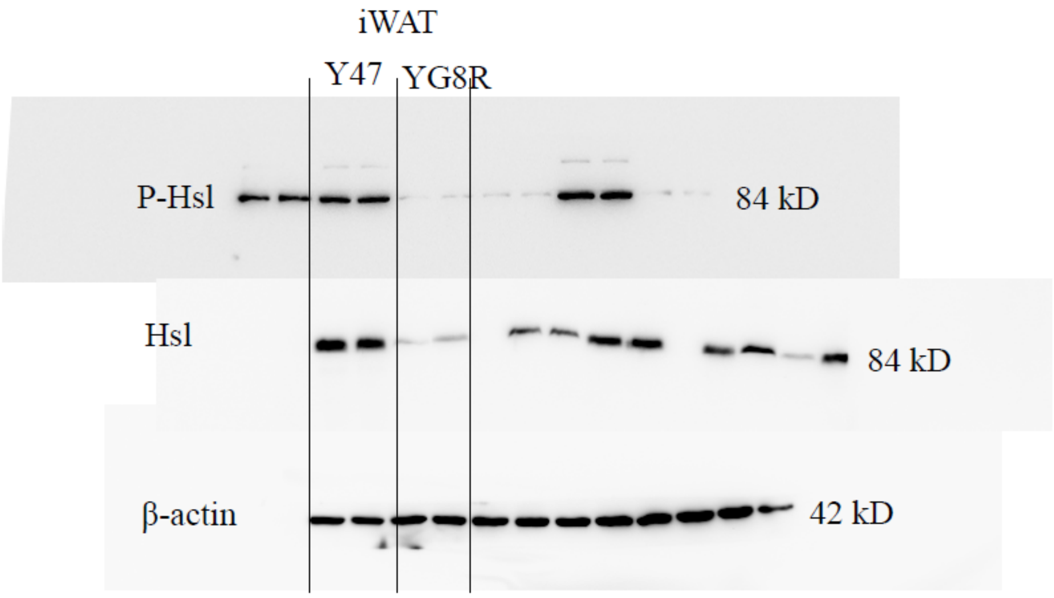

Figure S10. Uncropped images of immunoblots for figure 2I.

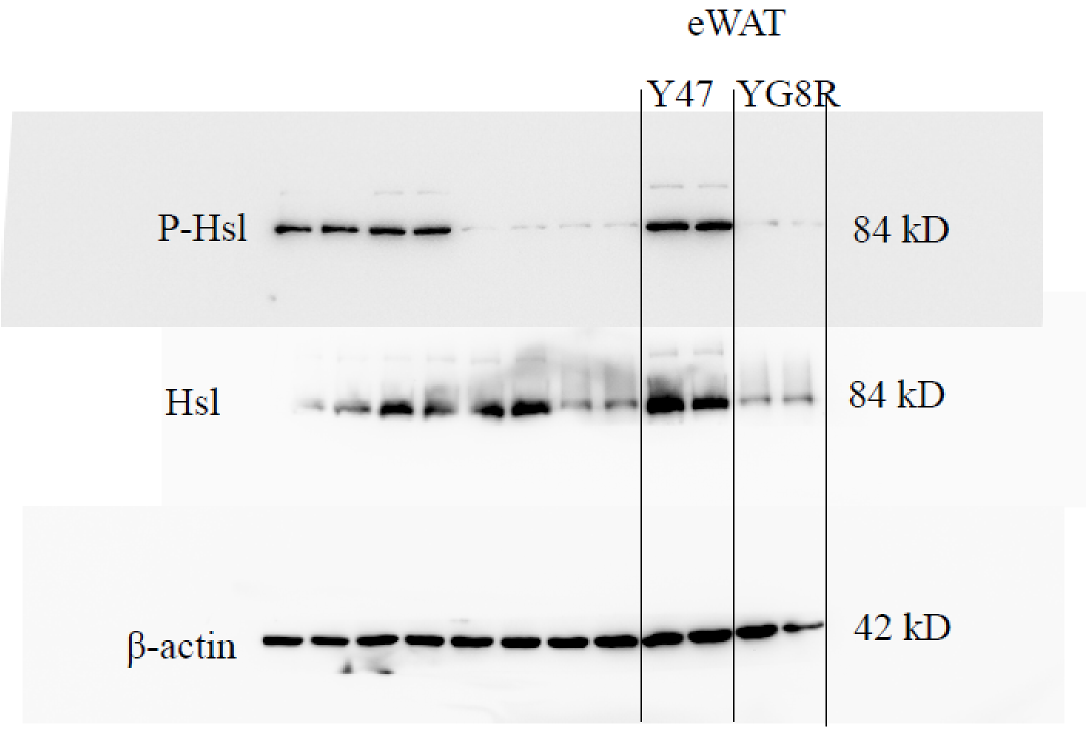

Figure S11. Uncropped images of immunoblots for figure 2I.

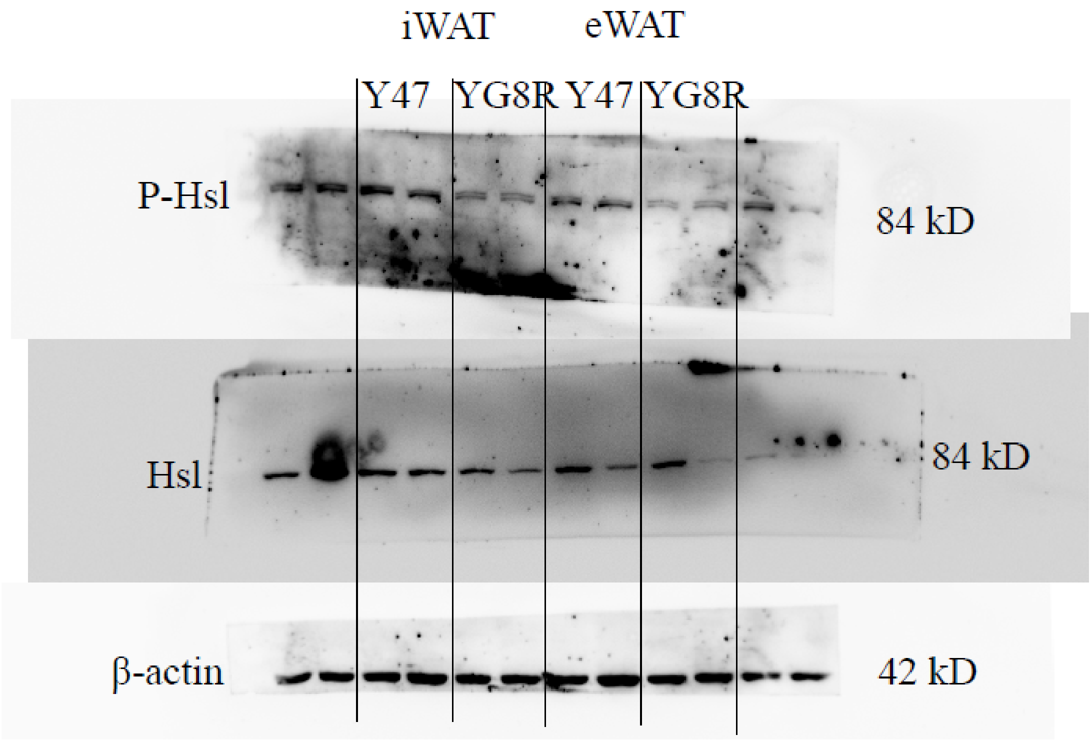

Figure S12. Uncropped images of immunoblots for figure 4M and 4N.

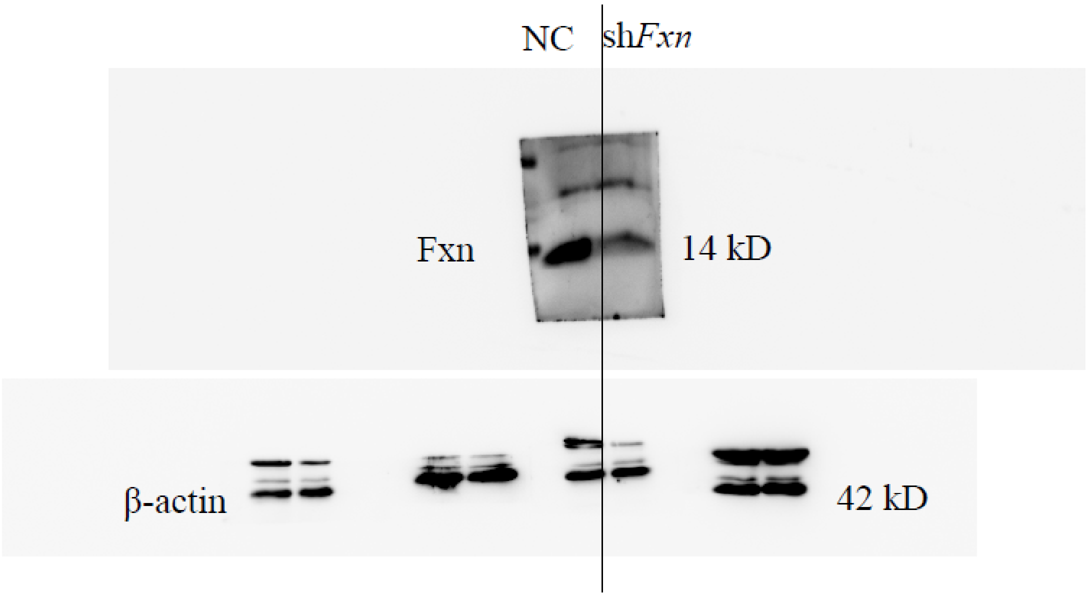

Figure S13. Uncropped images of immunoblots for figure 5A.

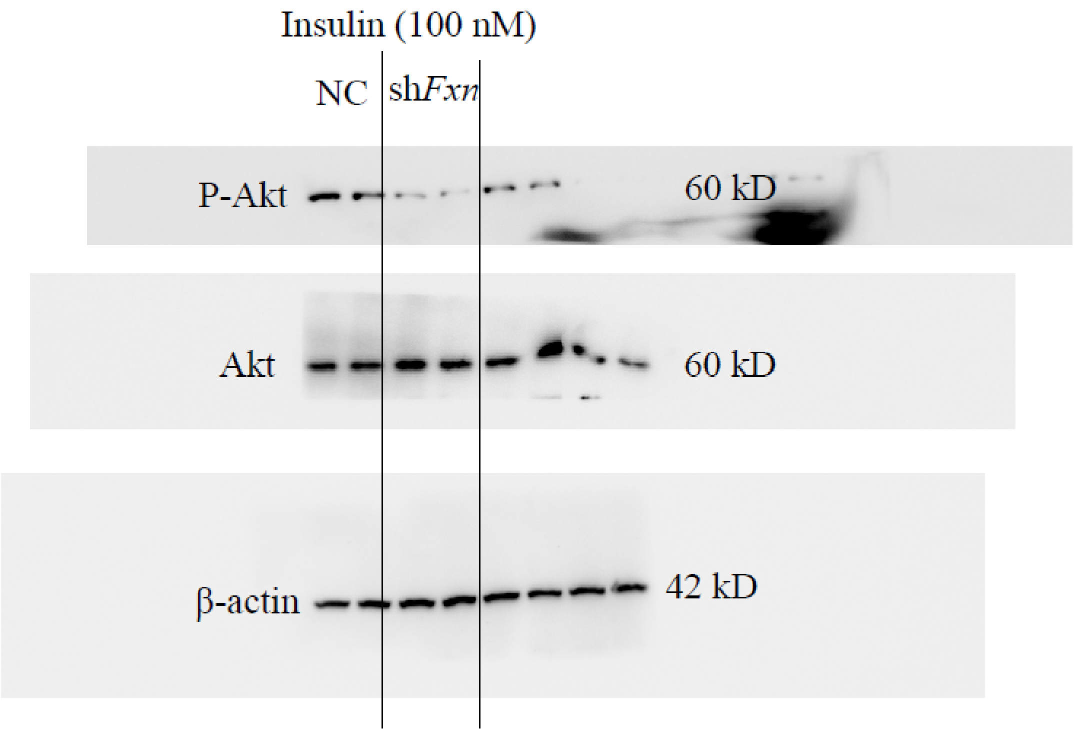

Figure S14. Uncropped images of immunoblots for figure 5F.

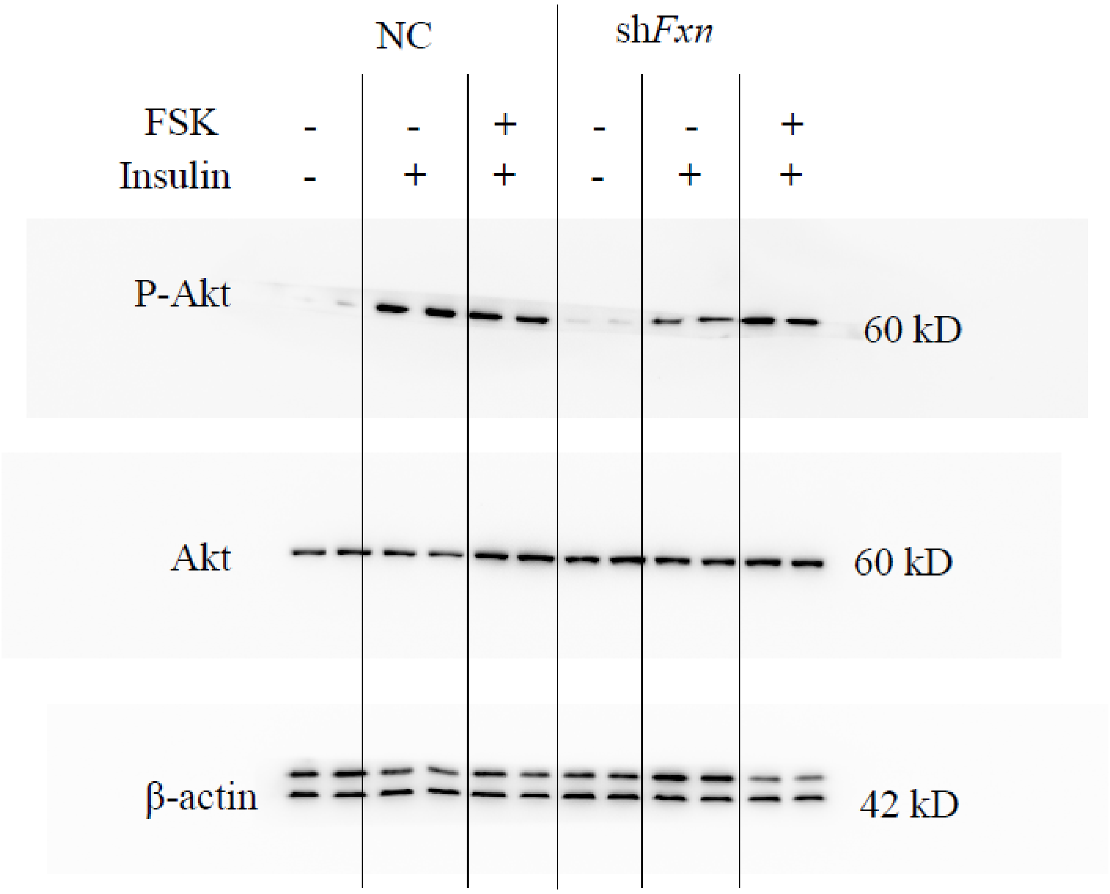

Figure S15. Uncropped images of immunoblots for figure 5I.

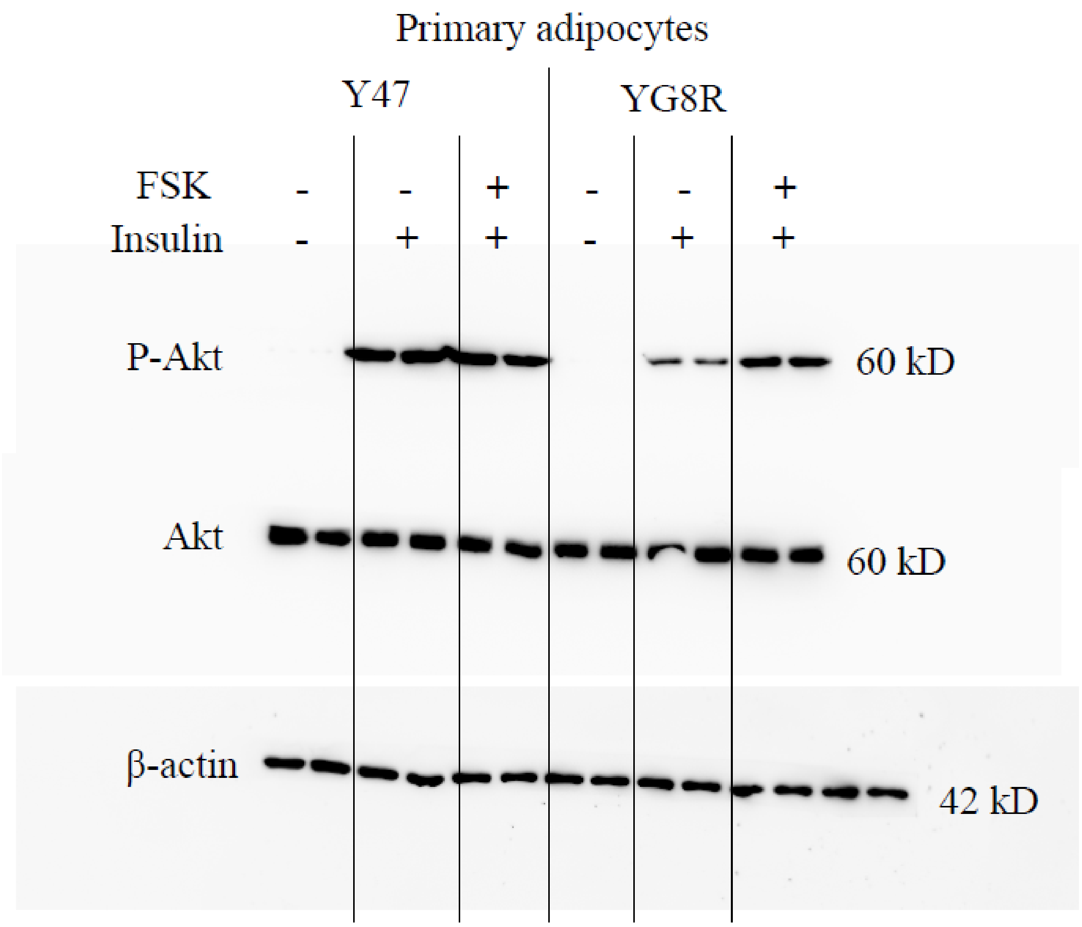

Figure S16. Uncropped images of immunoblots for figure 25J.

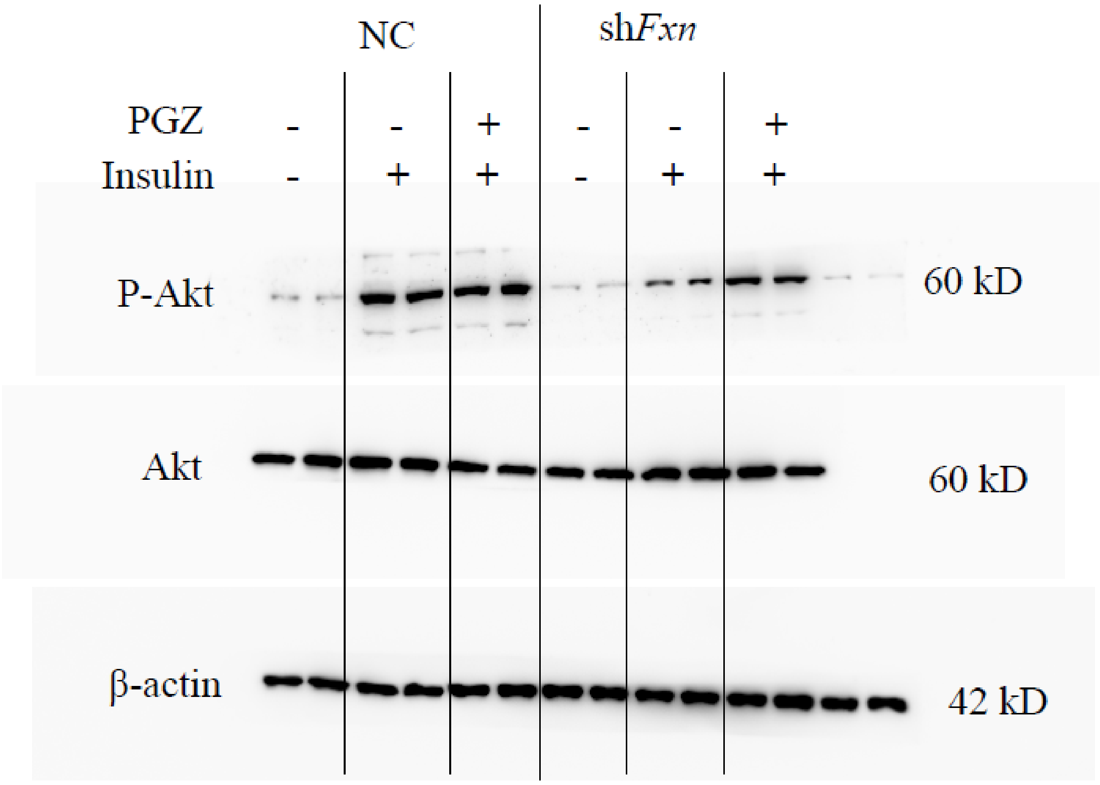

Figure S17. Uncropped images of immunoblots for figure 5K.

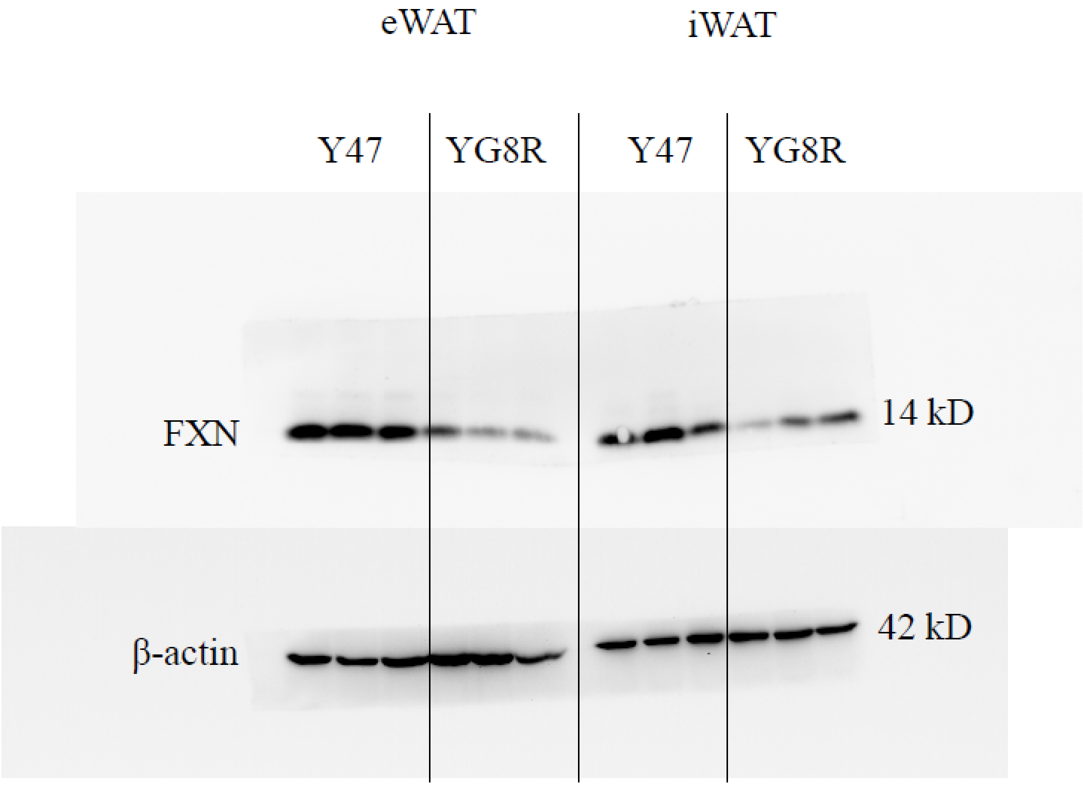

Figure S18. Uncropped images of immunoblots for figure S1A.

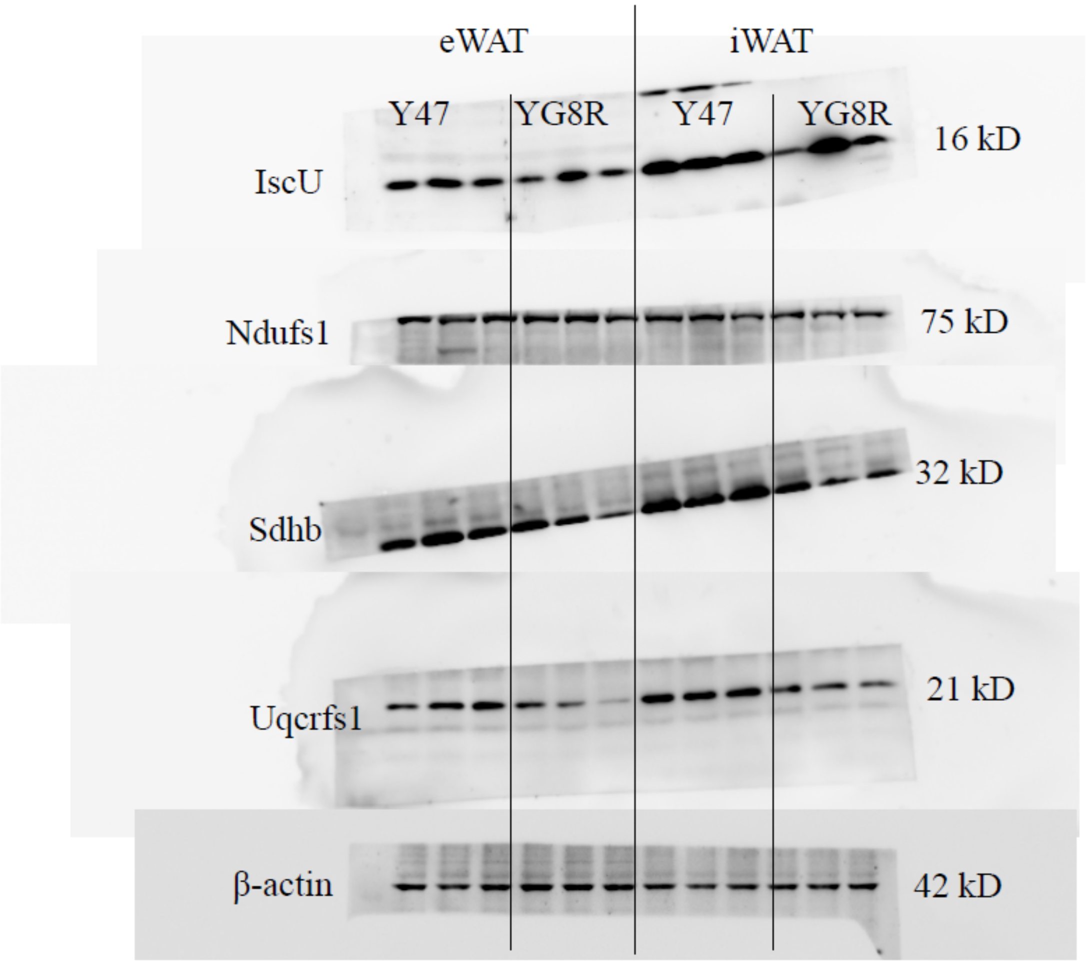

Figure S19. Uncropped images of immunoblots for figure S1B.

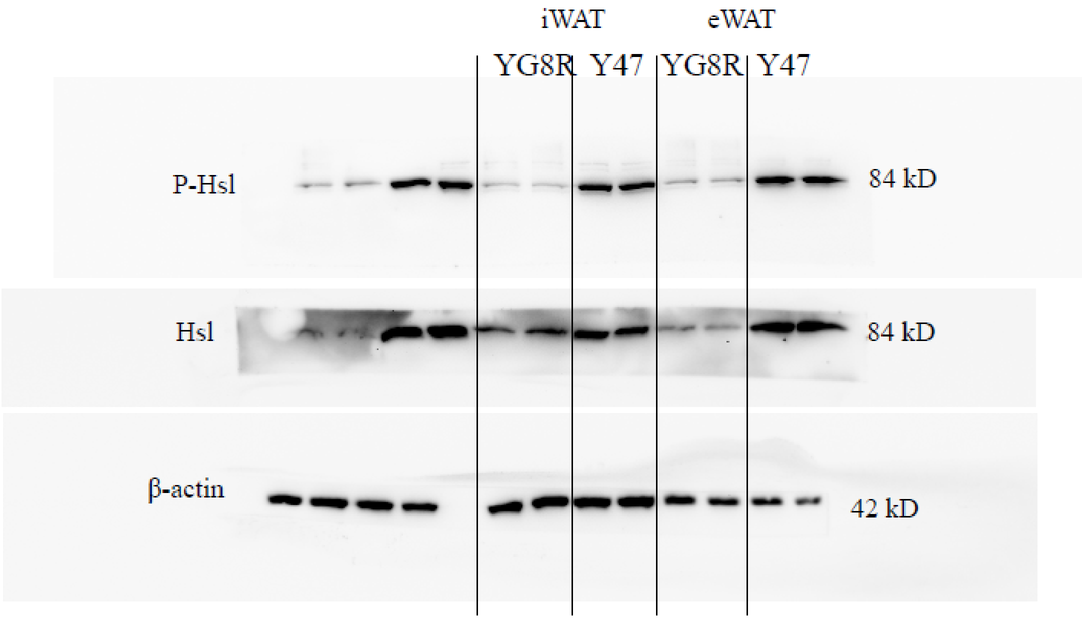

Figure S20. Uncropped images of immunoblots for figure S2F.
